# Long Term High‐Salt Diet Induces Cognitive Impairments via Down‐Regulating SHANK1

**DOI:** 10.1002/advs.202502099

**Published:** 2025-06-26

**Authors:** Cuiping Guo, Yuanyuan Li, Wensheng Li, Tongrui Wu, Yi Liu, Yacoubou Abdoul Razak Mahaman, Jianzhi Wang, Rong Liu, Wei Liu, Hui Shen, Xiaochuan Wang

**Affiliations:** ^1^ Institutes of Biomedical Sciences School of Medicine Hubei Key Laboratory of Cognitive and Affective Disorders Jianghan University Wuhan 430056 China; ^2^ Department of Pathophysiology School of Basic Medicine Key Laboratory of Education Ministry/Hubei Province of China for Neurological Disorders Tongji Medical College Huazhong University of Science and Technology Wuhan 430030 China; ^3^ Department of Emergency Medicine & Department of Critical Care Medicine, Tongji Hospital, Tongji Medical College Huazhong University of Science and Technology Wuhan 430030 China; ^4^ Laboratory of Neurobiology School of Basic medicine Tianjin Medical University Tianjin 300070 China; ^5^ Hubei Provincial Key Laboratory of Kidney Diseases Occurrence and Intervention School of Medicine Hubei Polytechnic University Huangshi Hubei Province 435003 China; ^6^ Co‐innovation Center of Neuroregeneration Nantong University Nantong 226001 China; ^7^ Department of Ophthalmology, Union Hospital, Tongji Medical College Huazhong University of Science and Technology Wuhan 430022 China

**Keywords:** high‐salt diet, SHANK1, PKA/CREB, synaptic dysfunction, cognitive deficits

## Abstract

High‐salt (HS) diet is an established risk factor for cognitive impairment, but the underlying mechanisms remain unclear. This study reveals that HS diet reduces SHANK1, a key postsynaptic scaffolding protein, via downregulation of the PKA/CREB pathway, leading to synaptic dysfunction and cognitive deficits in rats. RNA sequencing of HS‐fed rat hippocampi showed downregulation of cAMP signaling and SHANK1 expression. Pharmacological inhibition of PKA/CREB reduced SHANK1 levels and impaired dendritic structure and synaptic function, while PKA activation restored CREB activity and SHANK1 expression, reversing HS‐induced deficits. Notably, CREB activation is essential for SHANK1 regulation, as a CREB mutant (S133A) blocked the effects of PKA activation, and a constitutively active CREB (S133D) prevented SHANK1 downregulation. These findings highlight the PKA/CREB/SHANK1 pathway as a potential therapeutic target for HS‐induced cognitive dysfunction.

## Graphical abstract

The study identifies a novel mechanistic link between long‐term HS diet and cognitive impairment, wherein PKA/CREB axis inactivation leads to SHANK1 reduction, synaptic damage, and cognitive deficits.

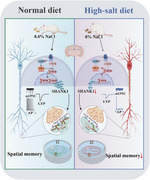

## 1 Introduction

High salt intake remains widespread globally, despite ongoing public health efforts to raise awareness of its associated health risks.^[^ ^1^, ^2^, ^3^ ^]^ Accumulating evidence from rodent models strongly suggests that 4–12 weeks of HS intake causes cognitive impairments, in addition to cardiovascular complications.^[^ ^4^, ^5^, ^6^, ^7^ ^]^ A 3‐year prospective study involving 1262 adults found that lower dietary sodium intake was associated with a reduced incidence of cognitive impairment.^[^ ^8^ ^]^ However, the precise molecular mechanisms underlying HS‐induced cognitive defects remain poorly understood.

HS diet is an independent risk factor for dementia and has been linked to cerebral small vessel disease, which underlies vascular cognitive impairment.^[^ ^4^, ^5^, ^9^ ^]^ Recent findings by Facaro et al. reported that the HS diet induces gut‐mediated adaptive immune activation, leading to increased IL‐17 levels and downregulation of endothelial nitric oxide synthase. These results in nitric oxide deficiency, decreased calpain nitrosylation, and subsequent activation of calpain and CDK5, promoting cognitive deficits via tau hyperphosphorylation.^[^ ^10^ ^]^ Interestingly, tau phosphorylation in the neocortex was observed to increase after 4 weeks of HS intake, whereas in the hippocampus, it was not evident until 12 weeks. Our previous studies demonstrated significant cognitive deficits following 8–9 weeks of chronic HS intake, yet these were not associated with tau phosphorylation, Aβ deposition, or neuronal loss in the hippocampus.^[^ ^11^ ^]^ Given that dementia, including Alzheimer's disease (AD), is characterized by synaptic dysfunction, formation of senile plaques, hyperphosphorylated tau tangles, neuroinflammation, and apoptotic cell death.^[^ ^12^, ^13^, ^14^, ^15^, ^16^, ^17^ ^]^ This suggests that synaptic dysfunction is preexisting in Aβ aggregation and tau pathology and may play a key role in HS diet‐induced cognitive impairments.

SHANK is a multidomain scaffold protein that mediates the integration of multiple synaptic proteins.^[^ ^18^, ^19^, ^20^ ^]^ The specific localization of SHANK proteins at postsynaptic sites of excitatory synapses in the brain suggests their roles in organizing cytoskeletal and signaling complexes at specialized cell junctions.^[^ ^18^, ^21^ ^]^ SHANK proteins are implicated in the pathophysiology of autism spectrum disorders and are encoded by the SHANK1, SHANK2, and SHANK3 genes.^[^ ^20^, ^21^, ^22^, ^23^, ^24^, ^25^ ^]^ However, the physiological role of SHANK proteins and their involvement in cognitive impairments, particularly in the context of HS diet, remains largely unexplored.

Based on the theoretical background and our previous findings, HS diet induces hippocampal‐dependent spatial memory impairment, accompanied by the inhibition of cAMP‐response element‐binding protein (CREB) activity and decreased phosphorylation at Ser133 of CREB.^[^ ^11^ ^]^ It is well‐established that members of the CREB family are key regulators of gene expression in response to cAMP signaling, via phosphorylation by protein kinase A (PKA). CREB is a nuclear protein that modulates the transcription of genes containing cAMP‐responsive elements within their promoters. Increased intracellular cAMP concentrations trigger the phosphorylation and activation of CREB, a key transcription factor that regulates numerous memory‐related processes.^[^ ^26^, ^27^, ^28^, ^29^, ^30^, ^31^ ^]^

In this report, we demonstrate that SHANK1 reduction leads to HS‐related cognitive deficits. Additionally, downregulation of SHANK1 exacerbates synaptic dysfunction and cognitive deficits, while upregulation of the PKA/CREB pathway increases SHANK1 expression, thereby improving synaptic damage and mitigating cognitive deficits. This study is the first to report on the dysfunction of the PKA/CREB/SHANK1 axis in HS diet‐induced learning and memory impairments, suggesting that enhancing PKA activity to increase SHANK1 levels may represent a promising therapeutic approach for treating HS‐induced cognitive dysfunction.

## 2 Results

### 2.1 High‐Salt Diet Induces Synaptic Dysfunction and Cognitive Impairments

**Figure 1:**
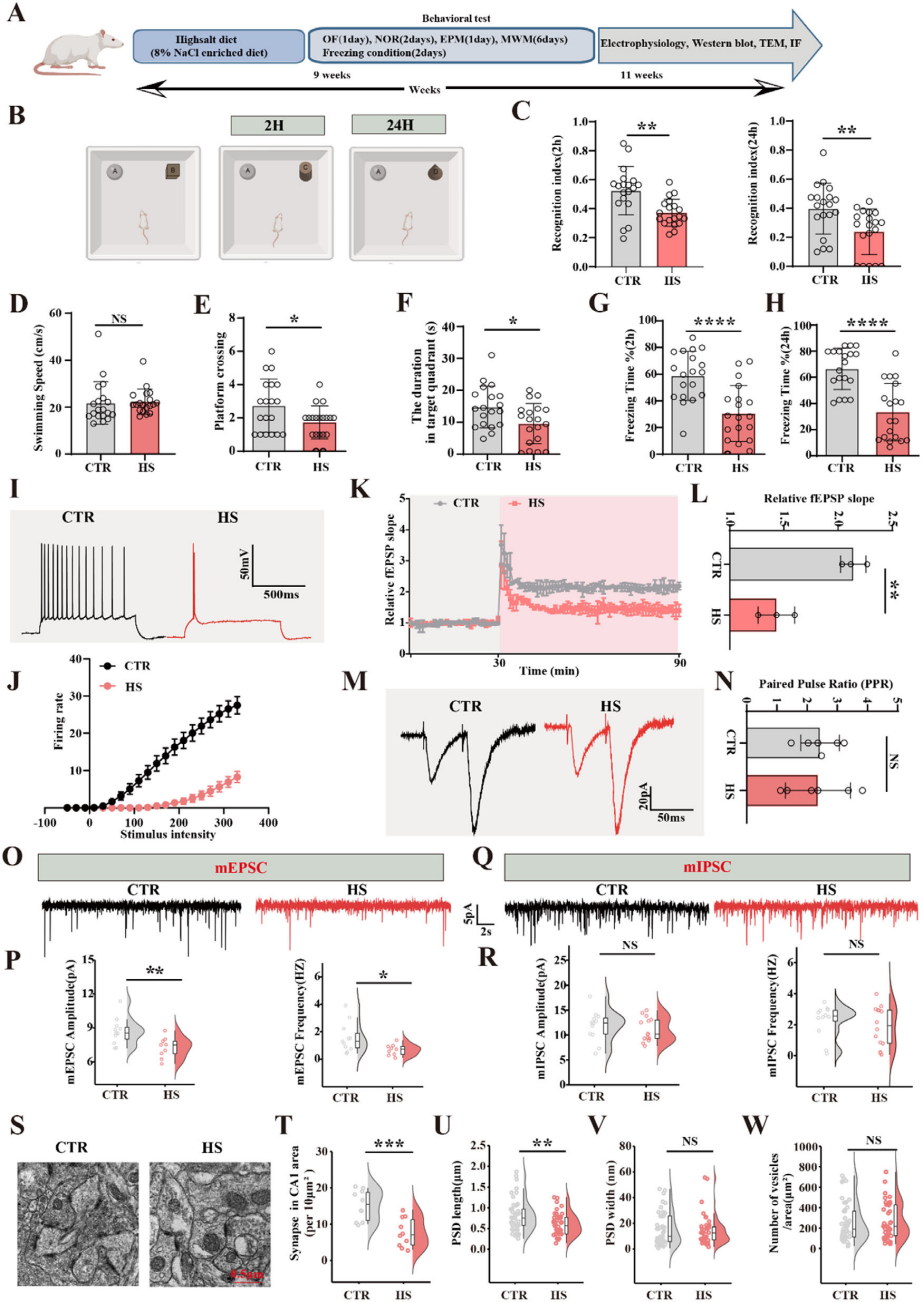
High‐salt diet induces synaptic dysfunction and cognitive impairments A) Experimental design schematic. Healthy male SD rats were randomly assigned to two groups: the control group received a normal diet, while the model group was subjected to HS diet (8% NaCl) for 9 weeks. Following dietary treatment, behavioral, electrophysiological, and biochemical assessments were conducted. B) The novel object recognition test showed the recognition index at 2 h and 24 h (n  = 19), (C). D–F) Morris water maze test: swimming speed (D), the spatial memory was tested on the 6th day by removing the platform the number of crossing the position of the target platform E) and the time spent in the target quadrant F) were measured, (n = 19). Fear conditioning test showed freezing duration was measured during the 3 min memory test at 2 h G) or 24 h H) after conditioning, (n  = 19). I, J) The firing rate of AP was measured, (n = 18–24 neurons from 3 rats). K) Hippocampal CA3‐CA1 LTP was recorded by the MED64 system. Normalized CA3‐CA1 fEPSP mean slope recorded from the CA1 dendritic region in acute hippocampal slices. Quantitative analyses for fEPSP measured 60–90 min after HFS relative to baseline L), (n = 3 slices from 3 rats). M) For pairwise pulse ratio experiments, paired stimuli with a 50 ms interval were delivered and the ratio was calculated as the glutamate receptor amplitude ratio (EPSC2 / EPSC1) N). Rat hippocampal neurons were subjected to whole‐cell patch‐clamp recording of mEPSCs O) amplitude and frequency of mEPSCs of control and HS neurons P), (n = 9–11 neurons from 3–4 rats). The miniature inhibitory postsynaptic potential (mIPSC) of the GAMA receptor was recorded by whole‐cell patch‐clamp Q), amplitude and frequency of mIPSCs of control and HS neurons R). (n = 12–14 neurons from 3–4 rats). S–W) The representative images showing transmission electron microscope (Scale bar, 0.5µm) (S), Quantitative analysis of the synaptic density (n = 10 from 3 rats per group) (T), the length of postsynaptic density (U), the width of postsynaptic denses (V) and the release of presynaptic vesicles (W) were measured (n  = 40–50 synapses from 3 rats). Data were expressed as mean ± SD, ^*^ *p* < 0.05, ^**^ *p* < 0.01, ^***^ *p* < 0.001, ^****^ *p* < 0.0001 vs control. Statistical details were provided in Supporting Information.

High salt is a well‐established risk factor for cognitive decline.^[^ ^7^, ^32^, ^33^ ^]^ In this study, 2‐month‐old SD rats were fed an HS diet (8% NaCl) for 9 weeks to establish an HS model.^[^ ^10^, ^11^ ^]^ Behavioral, electrophysiological, and biochemical analyses were performed (**Figure** **1**A). A series of behavioral tests were conducted to assess cognitive function. The open field test showed no significant differences in total distance covered (Figure S1A, Supporting Information) and the number of zone crossings (Figure S1B, Supporting Information) between groups, indicating that HS intake did not affect motor function. Social ability, assessed via the three‐chamber social test, revealed a significant reduction in the frequency of contact with unfamiliar rats in the HS group (Figure S1C, Supporting Information), though no differences were observed in the time spent in the novel box (Figure S1D, Supporting Information). Elevated plus maze (EPM) test results show that the HS diet significantly increased the open arm entry ratio without affecting the duration of the open arm ratio (Figure S1E,F,Supporting Information). These results suggest that a long‐term HS diet leads to anxiety and social disorders in rats.

To evaluate learning and memory, we performed the novel object recognition test, the Morris water maze (MWM), and fear conditioning experiments. In the novel object recognition test, rats in the HS group had a significantly reduced recognition index at 2 and 24 h for novel objects compared to controls (Figure 1B,C). The MWM results showed no differences in swimming speed between groups (Figure 1D). On day 6 after the platform was removed, HS rats exhibited significantly fewer platform crossings (Figure 1E) and spent less duration in the target quadrant (Figure 1F). In the fear conditioning test, freezing time in the HS group was significantly reduced at both 2 h (Figure 1G) and 24 h post‐conditioning (Figure 1H). These data demonstrate that the HS diet impairs memory in rats.

The hippocampus, a critical region of the brain involved in memory and spatial navigation, is essential for synaptic plasticity, the cellular basis of learning and memory.^[^ ^34^, ^35^, ^36^ ^]^ Anatomically, the hippocampus consists of distinct subregions: cornu ammonis area 1(CA1), cornu ammonis area 3(CA3), and the dentate gyrus (DG), with the CA1 region particularly vulnerable to injury and stress.^[^ ^34^, ^37^, ^38^ ^]^ To explore the mechanisms underlying HS diet‐induced cognitive deficits, we examined hippocampal CA1 neuronal activity, HS rats showed reduced action potential (AP) firing (Figure 1I,J). Additionally, we examined hippocampal CA1 synaptic plasticity. Long‐term potentiation (LTP) experiments showed that HS‐fed rats exhibited a significant reduction in the slope of field excitatory postsynaptic potentials (fEPSP) following high‐frequency stimulation (HFS) compared to controls (Figure 1K,L). To investigate the underlying synaptic transmission defects, the paired‐pulse ratio (PPR) was assessed to evaluate presynaptic vesicle release.^[^ ^39^, ^40^ ^]^ PPR analysis revealed no significant differences between the two groups at inter‐stimulus intervals of 50 ms (Figure 1M,N), as well as at 100 ms, and 150 ms (Figure S2A,B,Supproting Information). Whole‐cell patch‐clamp recordings were performed to measure miniature excitatory postsynaptic currents (mEPSC) (Figure 1O) and miniature inhibitory postsynaptic currents (mIPSC) (Figure 1Q) in the CA1 region. HS intake severely impaired excitatory synaptic transmission (Figure 1P) but had no effect on inhibitory synaptic transmission (Figure 1R). Moreover, transmission electron microscopy analysis revealed that the number of excitatory synapses per area in the CA1 region was significantly lower in HS‐fed rats compared to controls (Figure 1S, T). The length of the postsynaptic density (PSD) in individual excitatory synapses was also significantly reduced in the HS group (Figure 1U), with no significant changes in PSD width (Figure 1V) or synaptic vesicle count (Figure 1W). These findings confirm that a long‐term HS diet induces synaptic dysfunction and cognitive deficits.

**Figure 2:**
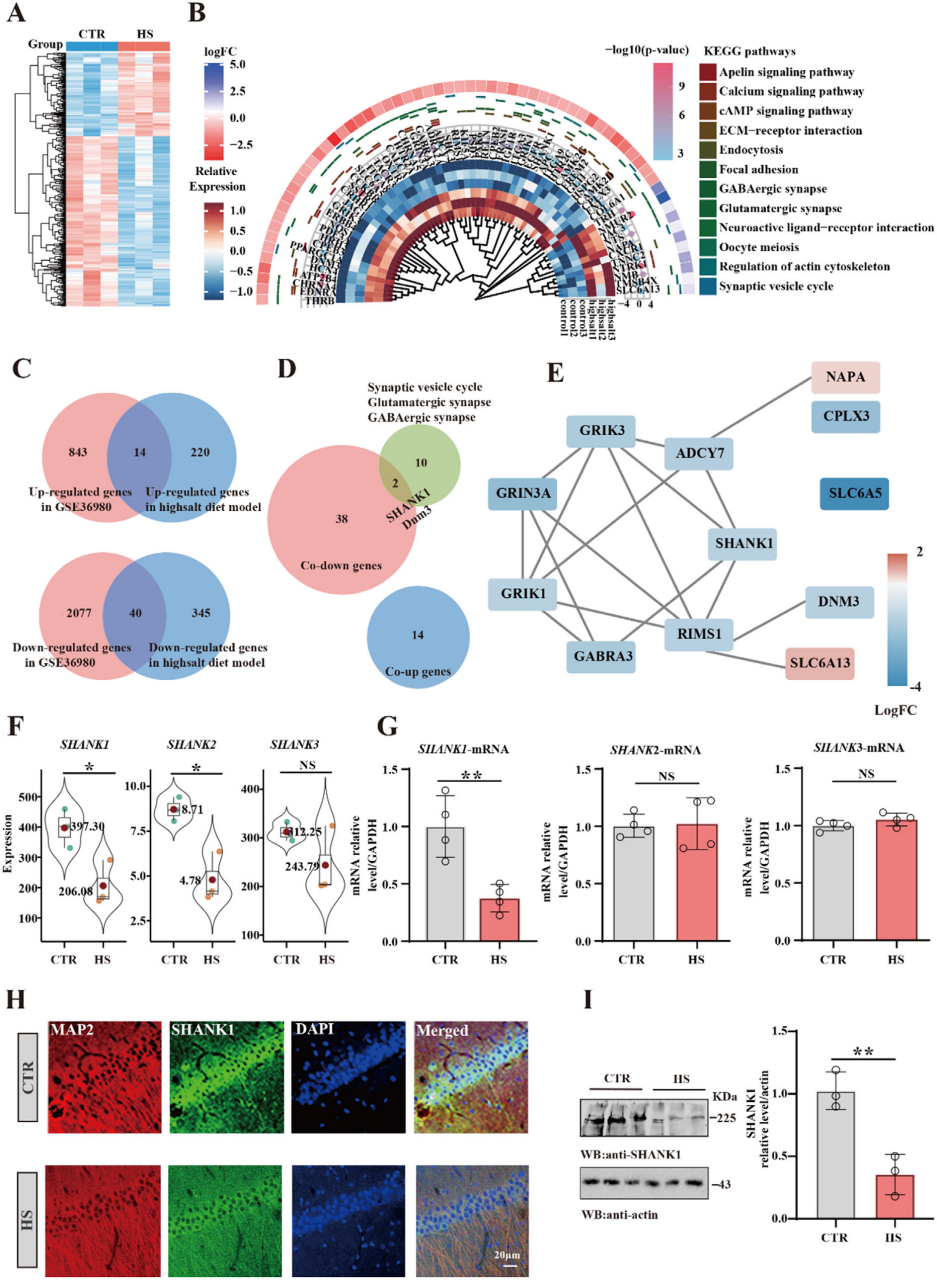
High‐salt diet significantly decreases the expression of SHANK1 A) Heatmap illustrating the expression levels of differentially expressed genes (DEGs) identified from RNA‐seq analysis of a HS diet rat model. B) KEGG pathway enrichment analysis results (|NES| > 0.5, p.adjust < 0.05, q‐value < 0.25). The core enrichment genes in the identified pathways are presented, including their average expression, logFC, and p‐value. C) Venn diagram showing the overlap between DEGs (p‐value < 0.05) in the GSE36980 dataset and RNA‐seq results from the HS diet rat model. A total of 14 co‐upregulated and 40 co‐downregulated genes were identified. D) Venn diagram illustrating the intersection between the co‐upregulated/downregulated genes identified in Figure 2C and the core enrichment genes in synapse‐related KEGG pathways (synaptic vesicle cycle, glutamatergic synapse, and GABAergic synapse pathways) in the HS diet rat model. SHANK1 and DNM3 were identified. E) PPI network of the core enrichment genes in the synaptic vesicle cycle, glutamatergic synapse, and GABAergic synapse pathways. F) SHANK1 /SHANK2/SHANK3 expression levels in RNA sequencing were measured. G)The mRNA levels of SHANK1 /SHANK2/SHANK3 were measured (n = 4). The immunofluorescence H) and Western blotting I) were measured to the SHANK1 level of the hippocampus CA1 (n = 3). Data were expressed as mean ± SD, ^*^ *p* < 0.05,^**^ *p* < 0.01 vs control. Statistical details were provided in Supporting Information.

### 2.2 High‐Salt Diet Significantly Decreases the Expression of SHANK1

To further investigate the molecular mechanisms underlying the synaptic transmission deficits observed in HS‐fed rats, we performed RNA sequencing analysis. A volcano plot (Figure S3A, Supporting Information) and heatmap (**Figure** **2**A) illustrate the differential expression of genes (DEGs) between the HS diet and control groups. Gene Ontology (GO) enrichment (Figure S3B, Supporting Information) and KEGG pathway analyses (Figure 2B, S3C,Supporting Information) revealed significant changes in synapse‐related pathways. Visualization of the top enriched cellular components and core enrichment genes identified from GO and KEGG analyses are shown (Figure S3D, E, Supporting Information). A Venn diagram comparing DEGs from our RNA‐seq data and the GSE36980 dataset revealed 14 co‐upregulated and 40 co‐downregulated genes (Figure 2C). We further examined the intersection between these co‐upregulated and co‐downregulated genes (identified in Figure 2C) and the core enrichment genes in synapse‐related KEGG pathways, specifically the synaptic vesicle cycle, glutamatergic synapse, and GABAergic synapse pathways, in the high‐salt (HS) diet rat model. This overlap was visualized in a Venn diagram, identifying SHANK1 and DNM3 as key genes (Figure 2D). The protein‐protein interaction (PPI) network of core enrichment genes in the synaptic vesicle cycle, glutamatergic synapse, and GABAergic synapse pathways (Figure 2E) shows that SHANK1 plays a central role. Our data showed that SHANK1 and SHANK2 are significantly reduced, but there were no differences in SHANK3 in RNA sequencing (Figure 2F). Quantitative PCR (qPCR) results corroborated these findings, demonstrating a significant reduction in SHANK1 mRNA levels, with no notable differences in SHANK2 and SHANK3 expression (Figure 2G). This was further confirmed by immunofluorescence analysis (Figure 2H) and western blotting (Figure 2I), both of which showed a marked decrease in SHANK1 protein levels in the hippocampal CA1 region of HS‐fed rats. These results suggest that HS diet specifically reduces SHANK1 expression, probably contributing to synaptic dysfunction in the hippocampus.

**Figure 3:**
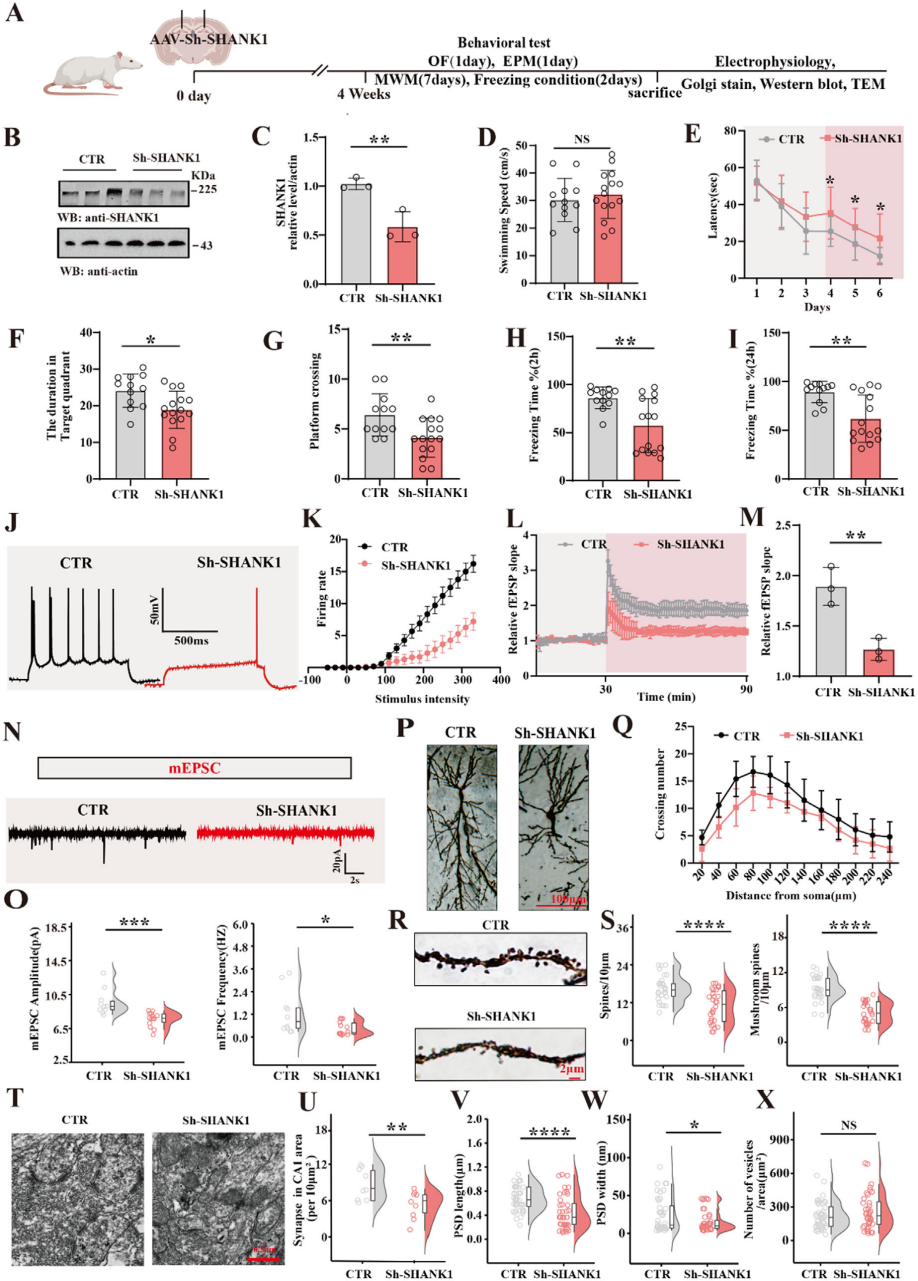
Down‐regulation of SHANK1 leads to synaptic damage and cognitive deficits A)Experimental design sketch. Healthy SD rats were randomly divided into 2 groups. In the control group, rats were injected with AAV/vector virus in the hippocampal CA1 region. In the model group, rats were injected with AAV‐Sh‐SHANK1 virus in the hippocampal CA1 region. Following the treatment, behavioral, electrophysiological, and biochemical tests were performed after 4 weeks. B) Brain tissues (hippocampus CA1 region) were homogenized, and SHANK1 protein levels were detected by immunoblotting. Actin was used as a loading control. Quantitative analysis of the SHANK1, (n = 3), (C). D–G) The Morris water maze test: the swimming speed (D) and the latency to find the hidden platform (E) from day 1 to day 6, the spatial memory was tested on the 7th day by removing the platform, and the time spent in the target quadrant (F) and the number of crossing the position of the target platform (G) were measured, (n = 12–15). (H, I) Fear conditioning test showed freezing duration was measured during the 3‐min memory test at 2 h H) or 24 h I) after conditioning, (n  = 12–15). J, K) The firing rate of action potential was reduced, (n = 18–24 neurons from 3 rats). L, M) Hippocampal CA3‐CA1 LTP was recorded by the MED64 system. Normalized CA3‐CA1 fEPSP mean slope recorded from the CA1 dendritic region in acute hippocampal slices. Quantitative analyses for fEPSP measured 60–90 min after HFS relative to baseline (n = 3 slices from 3 rats). Rat hippocampal neurons were subjected to whole‐cell patch‐clamp recording of mEPSCs N), amplitude and frequency of mEPSCs of control and HS neurons (O), (n = 10–14 neurons from 3 rats). P) The representative images of Golgi staining, and dendritic complexity were evaluated by sholl analysis,(n = 10 neurons from 3 rats), (Q). R) the density of dendritic spines (n = 30 neurons from 3 rats) and mushroom‐type spines (n = 30 neurons from 3 rats), (S) were tested. T–X) The representative images showing transmission electron microscope (Scale bar, 0.5µm) (T), Quantitative analysis of the synaptic density (n = 9 from 3 rats per group) (U), the length of postsynaptic density (V) and width of postsynaptic denses (W) and the release of presynaptic vesicles (X) were measured (n  = 40–45 synapses from 3 rats). Data were expressed as mean ± SD, ^*^ *p* < 0.05, ^**^ *p* < 0.01, ^***^ *p* < 0.001, ^****^ *p* < 0.0001 vs control. Statistical details were provided in Supporting Information.

### 2.3 Down‐Regulation of SHANK1 Leads to Cognitive Impairments and Synaptic Dysfunction

To evaluate the consequences of reduced SHANK1 in the hippocampus, we analyze the GSE36980 dataset. Bioinformatics analysis revealed that the expression of SHANK1 was reduced across various brain regions, including the frontal cortex (FC), temporal cortex (TC), and hippocampus (HP) in Alzheimer's disease (AD), based on the GSE36980 dataset (Figure S4A, Supporting Information). Using the GSE36980 dataset, correlation analysis further examined the expression levels of SHANK1, SHANK2, and SHANK3 in relation to age and gender (Figure S4B, Supporting Information). Regarding the issue of sex‐specific effects, we performed a sex‐stratified analysis within the GSE36980 dataset and results show that the correlation between SHANK1 expression and age is particularly strong in males (Figure S4C, Supporting Information). Since the GSE36980 dataset lacks detailed clinical and pathological data on Alzheimer's disease patients, we are unable to directly assess the correlation between SHANK1 and clinical disease severity within this dataset. Correlation analysis in GSE84422 reveals a negative association between SHANK1 expression and both neurofibrillary tangle density and clinical dementia rating, suggesting a potential link between SHANK1 levels and disease severity in Alzheimer's disease (Figure S4D, Supporting Information). To assess the functional consequences of SHANK1 downregulation on cognition, we injected AAV‐vector or AAV‐Sh‐SHANK1 bilaterally into the hippocampal CA1 region of 8‐week‐old SD rats. Behavioral, electrophysiological, and biochemical assessments were subsequently conducted (**Figure** **3**A). Western blotting confirmed a significant SHANK1 reduction in the Sh‐SHANK1 group (Figure 3B,C). A series of behavioral tests were performed to evaluate cognition. The open field test showed no significant differences in total distance traveled (Figure S5A, Supporting Information) or zone crossing (Figure S5B, Supporting Information) between the control and Sh‐SHANK1 groups. However, in the elevated plus maze test, the Sh‐SHANK1 rats exhibited a significant increase in the open arm entry ratio without affecting the duration in the open arm ratio (Figure S5C,D, Supporting Information), indicating heightened anxiety‐like behavior. Cognitive function was assessed using the Morris water maze and fear conditioning tests. In the Morris water maze, Sh‐SHANK1 rats demonstrated significantly longer latency to find the hidden platform during training (Figure 3E), with no significant differences in swimming speed (Figure 3D). On the probe trial, time spent in the target quadrant (Figure 3F) and platform crossing (Figure 3G) were significantly reduced in the Sh‐SHANK1 group. In the fear conditioning test, freezing time was markedly decreased in the Sh‐SHANK1 group at both 2 h (Figure 3H) and 24 h (Figure 3I), indicating impaired memory deficits.

To further investigate the role of SHANK1 deficiency in the induction of learning and memory impairment. SHANK1 knockdown significantly diminished AP firing rates (Figure 3J,K), which suggests SHANK1 downregulation reduced neuronal activity. Next, we evaluated the effect of SHANK1 knockdown on synaptic function. Electrophysiological recordings showed that the Sh‐SHANK1 rats exhibited a significantly reduced slope of fEPSP following HFS, compared to control rats (Figure 3L,M). Whole‐cell patch‐clamp recordings in the CA1 region revealed a substantial reduction in both the amplitude and frequency of mEPSC in Sh‐SHANK1 rats (Figure 3N,O). Golgi staining further demonstrated reduced dendritic complexity in Sh‐SHANK1 neurons, as evidenced by Sholl analysis (Figure 3P, Q), along with decreased spine density and a reduction in the mature mushroom‐type spines (Figure 3R, S). Transmission electron microscopy confirmed a significant reduction in the number of excitatory synapses per unit area in the CA1 region (Figure 3T,U), as well as a decrease in both the length (Figure 3V) and width (Figure 3W) of the postsynaptic density. However, no significant differences were observed in the number of synaptic vesicles between the two groups (Figure 3X). Additionally, primary hippocampal neurons were transfected with AAV‐GFP‐vector or AAV‐GFP‐Sh‐SHANK1 (Figure S6A, Supporting Information). AAV/GFP labeling revealed a reduction in dendritic complexity and total dendritic length in Sh‐SHANK1 neurons compared to controls (Figure S6A–C, Supporting Information), suggesting that SHANK1 deficiency results in morphological abnormalities of neurons. In summary, these findings strongly demonstrate that SHANK1 downregulation leads to synaptic damage and cognitive impairments, further implicating SHANK1 as a critical player in synaptic and cognitive function.

### 2.4 High‐Salt Diet Downregulates the PKA/CREB Signaling Pathway, Leading to SHANK1 Reduction

**Figure 4:**
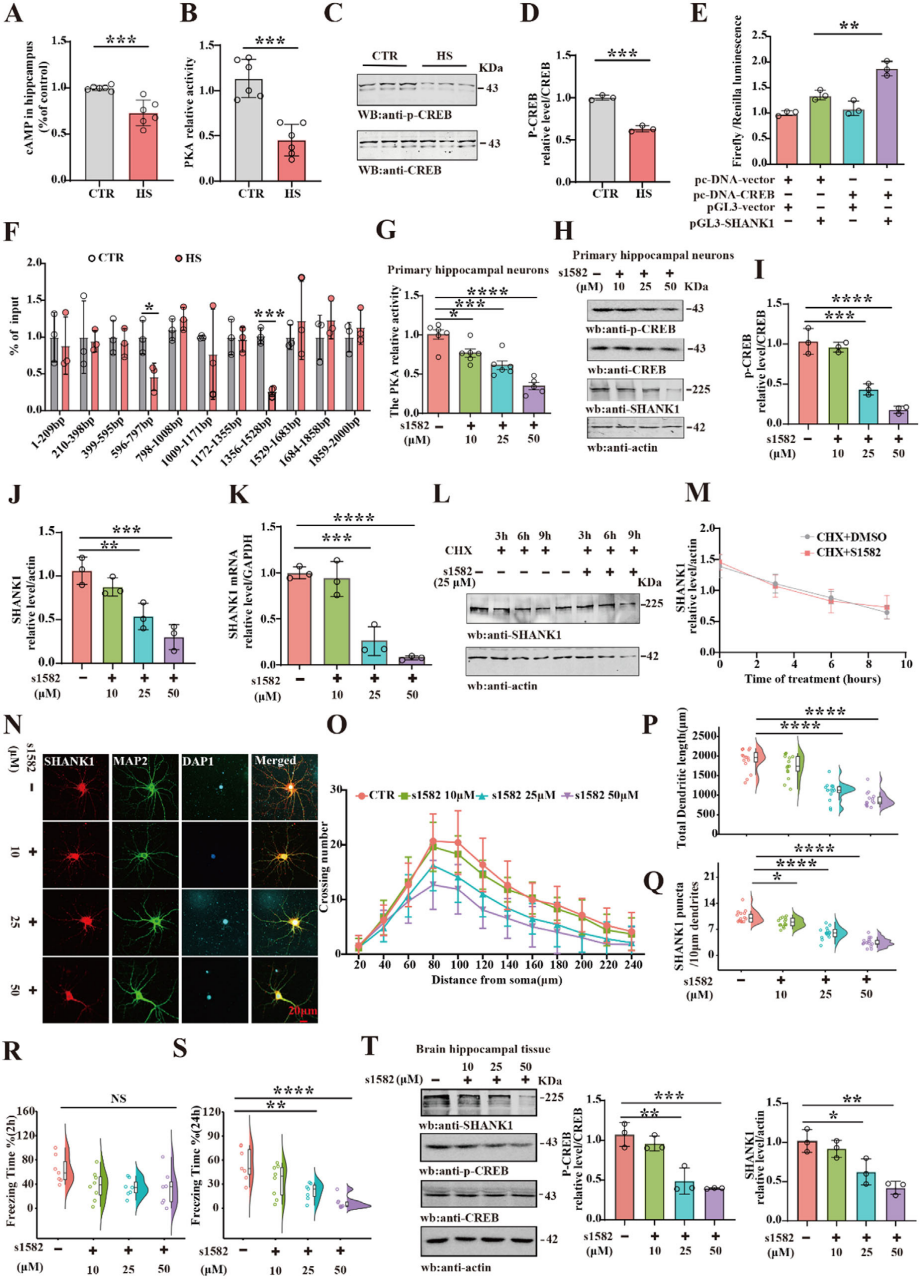
A) High‐salt diet downregulated the PKA/CREB signaling pathway, leading to SHANK1 reduction The level of cAMP was detected by Elisa Kit (n = 6). B) The PKA kinase activity was detected by Elisa Kit (n = 6). C) Brain tissues (hippocampus CA1 region) were homogenized, and pCREB/CREB protein levels were detected by immunoblotting. Actin was used as a loading control. Quantitative analysis of the pCREB/CREB, (n = 3), (D). E) The dual luciferase gene report assay was tested to the relationship between the CREB and SHANK1, (n = 3). F) Chromosome immunoprecipitation assay was showed that the relationship of CREB and SHANK1, (n = 3). G) Primary hippocampal neurons were treated with PKA inhibitor s1582 at 10, 25, and 50 µm. The PKA activity was measured, (n = 5–6). Western blotting H) was performed by using antibodies against SHANK1, CREB, and p‐CREB. Quantitative analysis of the protein levels (I, J) (n = 3). K) mRNA levels of SHANK1 were measured, (n = 3). (L, M) After the PKA inhibitor was administered to hippocampal primary neurons, cycloheximide (CHX) was added for 3 h, 6 h, and 9 h later, the degradation of SHANK1 was detected by western blotting (L), and quantitative analysis of the degradation level, (n = 3), (M). N) Immunofluorescence showed the representative images from hippocampal primary neurons treated with s1582 10, 25, and 50 µm for 24 h. Sholl analysis of dendrite complexity(O), (n = 10 neurons), quantification for the dendritic length (P) and the SHANK1 puncta/10µm dendrites (Q) (n = 15 neurons). By injection s1582 into hippocampus CA1, Fear condition was used to measure the contextual memory: freezing time during the 3 min memory test done at 2 h (R) or 24 h (S), (n = 7–8). T) The levels of CREB, p‐CREB, SHANK1 proteins were detected by western blotting, and quantitative analysis of the protein levels, (n = 3). Data were expressed as mean ± SD, ^*^ *p* < 0.05, ^**^ *p* < 0.01, ^***^ *p* < 0.001, ^****^ *p* < 0.0001vs Control or s1582. Statistical details were provided in Supporting information.

Our previous research demonstrated that HS diet induced hippocampal‐dependent spatial memory impairments, accompanied by decreased CREB phosphorylation at Ser133, and inhibited CREB activity.^[^ ^11^ ^]^ RNA sequencing analysis suggested that the HS diet suppressed the cAMP pathway (Figure 2B). It is well‐established that members of the CREB family are pivotal regulators of gene expression in response to cAMP, following phosphorylation by cAMP‐dependent protein kinase (PKA).^[^ ^41^ ^]^ However, it is not clear whether and how PKA/CREB pathway abnormalities and SHANK1 deficiency co‐contribute to cognitive dysfunction caused by HS diet. Therefore, we first investigated the cAMP/PKA signaling and found significantly lower cAMP levels in the hippocampus of HS rats compared to controls (**Figure** **4**A), alongside reduced PKA kinase activity (Figure 4B). Western blotting also confirmed a significant reduction in phosphorylated CREB‐Ser133 (p‐CREB) in the HS group (Figure 4C, D). Since cAMP elevation promotes PKA activation and induces CREB phosphorylation into the nucleus, which regulates gene transcription and is critical for memory function, CREB and SHANK1 mRNA levels are significantly reduced in the hippocampus of HS rats, we hypothesize that HS‐induced cognitive impairment and reduced SHANK1 are mediated by reduced CREB transcriptional activity in the cAMP/PKA pathway. Using JASPAR software, CREB is predicted to be a potential transcription factor for SHANK1 (Figure S7, Supporting Information). Dual‐luciferase reporter assays confirmed a positive regulatory relationship between CREB and SHANK1 (Figure 4E). To further verify this, chromatin immunoprecipitation (ChIP) assays were performed, revealing reduced CREB binding to sites 4 (596–797 bp) and 8 (1356–1528 bp) within the 2000‐bp upstream non‐coding region of SHANK1 in HS rats, compared to controls (Figure 4F). These results suggest that HS diet inhibits CREB activity, leading to reduced SHANK1 transcription.

To investigate the regulation of SHANK1 by the PKA/CREB pathway in vitro, primary hippocampal neurons were treated with s1582, a PKA inhibitor. The CCK‐8 assay was used to screen the effect of s1582 on cells and establish the concentration gradient. The results are presented in Figure S8 (Supporting Information). A significant decrease in cell viability was observed in the group treated with 100 µm s1582 at 24 h. Therefore, we chose 10, 25, and 50 µm concentrations to treat primary hippocampal neurons for 24 h. s1582 significantly decreased PKA kinase activity in dose‐dependent manner (Figure 4G) and reduced CREB Ser133 phosphorylation and SHANK1 protein levels (Figure 4H–J), as well as SHANK1 mRNA expression (Figure 4K). This suggests that inhibition of PKA downregulates the CREB/SHANK1 pathway. Additionally, to assess whether PKA inhibition affected SHANK1 protein degradation, primary hippocampal neurons were treated with cycloheximide (CHX) following s1582 treatment. Western blotting showed that s1582 did not influence SHANK1 degradation (Figure 4L, M). s1582 treatment significantly decreased dendritic complexity (Figure 4N, O), total dendritic length (Figure 4P), and SHANK1 puncta in hippocampal neurons (Figure 4Q). Additionally, we bilaterally injected the s1582 into the hippocampal CA1 region in 2‐month‐old male SD rats, and behavioral testing commenced 48 h post‐injection. When s1582 was injected into the hippocampal CA1 region in SD rats at concentrations of 10, 25, and 50 µm (2µL), PKA kinase activity was significantly reduced (Figure S9A, Supporting Information). Behavioral assays revealed that the open field test showed no significant differences in total distance covered (Figure S9B, Supporting Information) and the reduced number of zone crossings at s1582 50 µm (Figure S9C, Supporting Information). The novel object recognition test showed a significant reduction in the recognition index in the s1582‐treated group at 24 h (Figure S9E, Supporting Information), without change at 2 h (Figure S9D, Supporting Information). The fear conditioning test indicated no change in freezing behavior at 2 h post‐conditioning (Figure 4R), but a significant reduction was observed at 24 h (Figure 4S). Moreover, s1582 injected into the hippocampal CA1 region in SD rats led to a significant dose‐dependent decrease in SHANK1 protein expression and phosphorylated CREB (Figure 4T). Collectively, these findings suggest that inhibition of PKA/CREB pathway leads to SHANK1 reduction, which in turn may contribute to synaptic and cognitive dysfunction.

### 2.5 PKA Activation Promotes SHANK1 Expression and Rescues HS‐Related Cognitive Impairments

**Figure 5:**
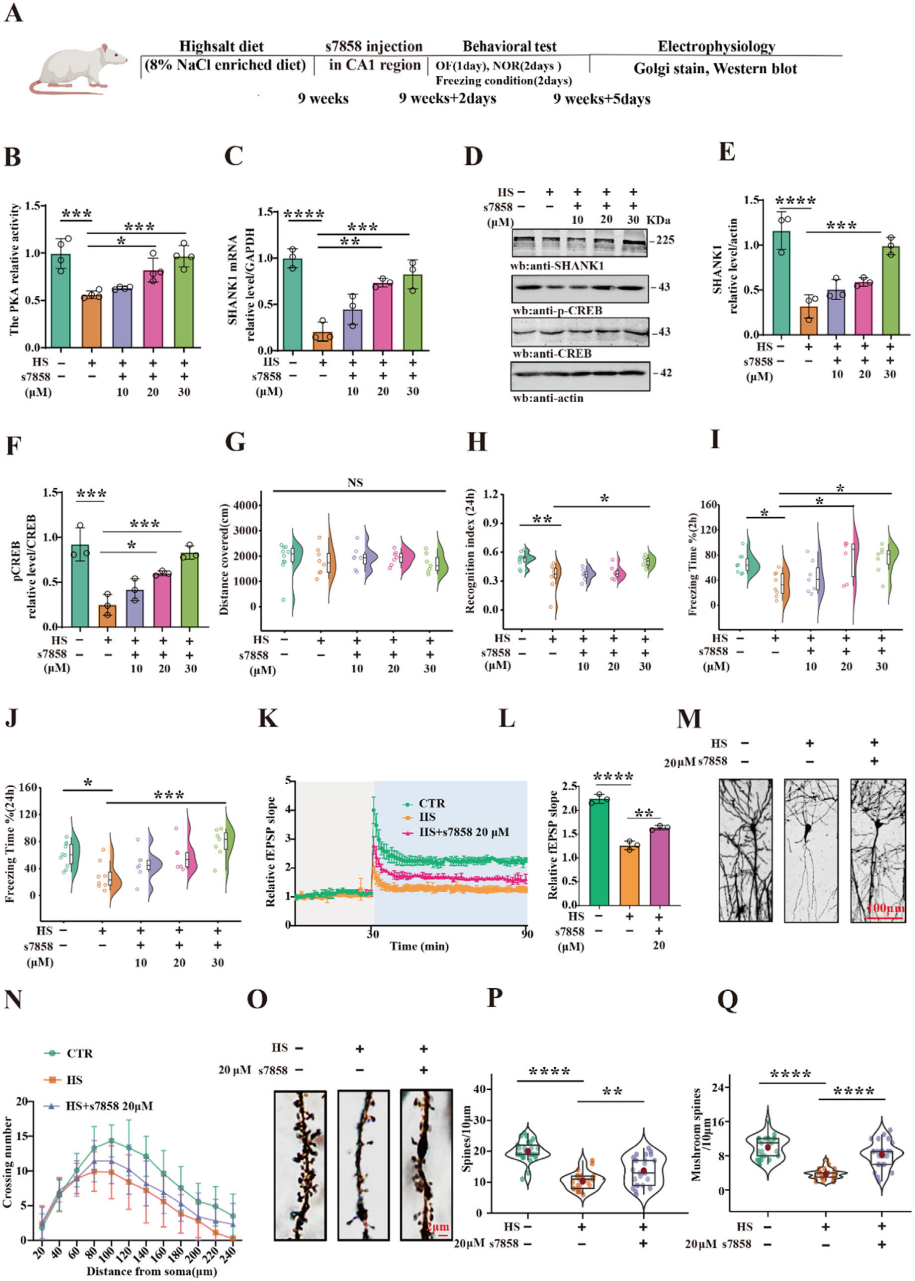
PKA activation promotes SHANK1 expression and rescues HS‐related cognitive impairments A) Experimental design sketch. After 9 weeks HS diet, s7858(PKA agonist) 2µL (10, 20, 30 µm) were injected into hippocampal CA1. B) The PKA kinase activity was detected by Elisa Kit, (n = 4). C) mRNA levels of SHANK1 were measured by qPCR,(n = 3). D–F) Brain tissues (hippocampus CA1 region) were homogenized, SHANK1 and pCREB/CREB levels were detected by immunoblotting. Actin was used as a loading control. Quantitative analysis of the SHANK1 (E) and pCREB/CREB (F), (n = 3). G) Open field test showed spontaneous exercise(n = 7–10). H) The novel object recognition test showed the recognition index at 24 h,(n = 7–10). I,J) Fear condition was performed to assess: Freezing time during the 3‐min memory test at 2 h (I) or 24 h,(n = 7–10), (J). K, L) Hippocampal CA3‐CA1 LTP was recorded by the MED64 system. Normalized CA3‐CA1 fEPSP means slope recorded from the CA1 dendritic region in acute hippocampal slices. (L) Quantitative analyses for fEPSP measured 60–90 min after HFS relative to baseline (n = 3 slices from 3 rats). M) Golgi staining was performed to show spines density and maturation in the hippocampal CA1 region. N) The representative images of dendritic trees in the CA1 of rats in three groups, (bar = 100 µm). Sholl analysis (N) and dendritic complexity analysis were performed to evaluate the dendritic complexity (n = 10 neurons from 3 rats). O) Representative images of dendritic spines. P) The quantitative analysis for the spine density (per 10 µm) and Q) percentage of mushroom‐type spines (n = 25 neurons from 3 rats). Data were expressed as mean ± SD, ^*^ *p* < 0.05, ^**^ *p* < 0.01, ^***^ *p* < 0.001, ^****^ *p* < 0.0001 vs HS group. Statistical details were provided in Supporting Information.

The above findings indicate that the reduction of SHANK1 is involved in HS diet‐induced synaptic dysfunction and cognitive deficits through PKA inactivation, suggesting that PKA activation may potentially mitigate HS‐related cognitive impairments. To test this hypothesis, 8‐week‐old male SD rats were fed with HS diet for 9 weeks, followed by bilateral hippocampal CA1 injections of s7858, a PKA agonist, at concentrations of 10, 20, and 30 µm (2µL). Behavioral, electrophysiological, and biochemical assessments were subsequently performed (**Figure** **5**A). s7858 administration significantly elevated PKA activity (Figure 5B), as well as SHANK1 mRNA and protein levels (Figure 5C–E), and phosphorylation of CREB at Ser133 (Figure 5D, F) in the hippocampus of HS rats. These findings further support that PKA/CREB activation upregulates SHANK1 transcription. Behavioral tests revealed no significant differences between groups in the open field test (Figure 5G). However, in the novelty recognition test, the HS + s7858 group exhibited a significantly higher recognition index for novel objects compared to the HS group at 24 h (Figure 5H). Moreover, fear conditioning tests showed that freezing behavior in the HS + s7858 group was significantly increased at both 2 h (Figure 5I) and 24 h (Figure 5J), compared to the HS group. Electrophysiological studies further confirmed that PKA activation enhanced the slope of fEPSP following high‐frequency stimulation relative to the HS group (Figure 5K, L). Golgi staining analysis also showed S7858 can alleviate HS‐induced dendritic damage, as assessed by Sholl analysis, in the HS + s7858 group (Figure 5M, N). Additionally, PKA activation elevated dendritic spine density and mushroom‐type spines (Figure 5O–Q). These findings strongly support the hypothesis that PKA activation promotes SHANK1 expression and rescues cognitive deficits and synaptic dysfunction caused by the HS diet.

### 2.6 CREB is Essential for PKA Regulation of SHANK1 Expression and Neuronal Dendritic Growth

**Figure 6:**
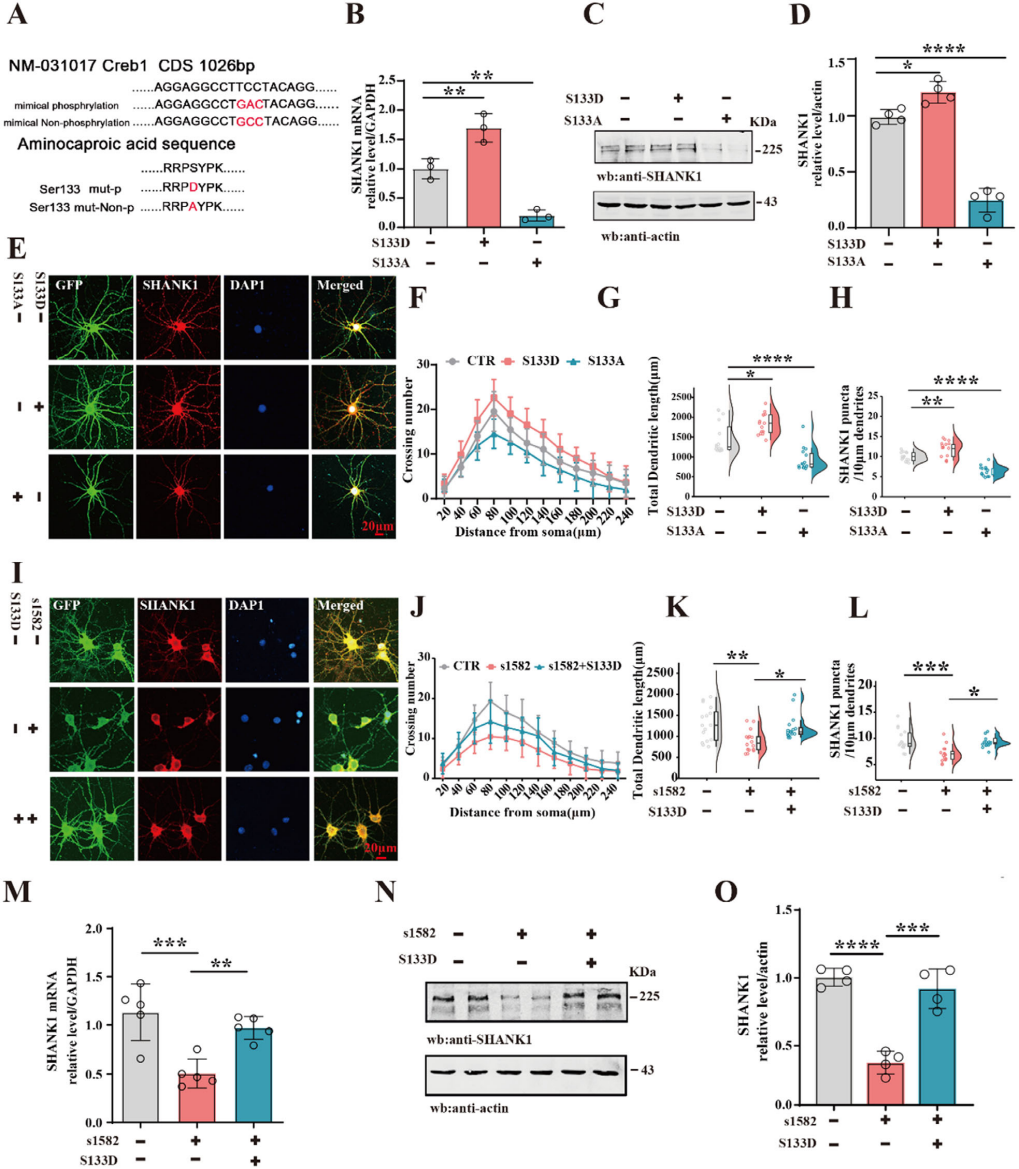
CREB is essential for PKA regulation of SHANK1 expression and neuronal dendritic growth A) The two mutated viruses (AAV‐CREB‐S133D/S133A) were transfected in primary hippocampal neurons. The SHANK1 mRNA was measured by qPCR, (n = 3) (B). C) Western blotting was employed to detect the SHANK1 protein level, actin was used as a loading control. Quantitative analysis of SHANK1 (D), (n = 4). E) Representative immunofluorescence images showed the total length of dendrites in neurons. Scale bar = 20 µm), Sholl analysis (F), quantitative analyses of dendritic length G) and the SHANK1 puncta/10µm dendrites H) (n = 15 neurons). I) Primary hippocampal neurons were treated with PKA inhibitor/s1582 and AAV‐CREB‐S133D, representative immunofluorescence images were shown (Scale bar = 20 µm), Sholl analysis J), quantitative analyses of dendritic length K) and the SHANK1 puncta/10µm dendrites (L), (n = 15‐16 neurons). M) The SHANK1 mRNA was measured by qPCR, (n = 5). N)Western blotting was employed to detect the SHANK1 protein levels, actin was used as a loading control. Quantitative analysis of SHANK1 O), (n = 4). All data represent mean ± SD, ^*^ *p* < 0.05, ^**^ *p* < 0.01, ^***^ *p* < 0.001, ^****^ *p* < 0.0001 vs Control or s1582 group. Statistical details were provided in Supporting Information.

To further elucidate the role of CREB in the transcriptional regulation of SHANK1 and its impact on synaptic function, we employed two CREB mutations: a phosphorylation mimic (S133D, which simulates an activated state) and a non‐phosphorylation mimic (S133A, which simulates an inactivated state) at the Ser133 site (**Figure** **6**A). These mutated CREB constructs were transfected into primary hippocampal neurons. qPCR analysis revealed that CREB‐S133D significantly upregulated SHANK1 expression, whereas the S133A mutation suppressed SHANK1 mRNA levels (Figure 6B). Western blotting further confirmed these findings, showing increased SHANK1 protein levels in the CREB‐S133D group, while SHANK1 expression was reduced in the CREB‐S133A group (Figure 6C, D).

To examine the impact of these mutations on dendritic morphology, primary hippocampal neurons were transfected with AAV‐GFP‐CREB‐S133D/S133A (Figure 6E), and GFP labeling was used to visualize dendritic structure. CREB‐S133D significantly enhanced dendritic complexity (Figure 6F), total dendritic length (Figure 6G), and SHANK1 puncta (Figure 6H), while CREB‐S133A reduced dendritic complexity and SHANK1 expression (Figure 6F‐H). In line with this, primary hippocampal neurons were treated with PKA inhibitor/s1582 and AAV‐CREB‐S133D. Immunofluorescence results also showed that the dendritic complexity, total dendritic length, and SHANK1 puncta were significantly increased in the s1582 + S133D group compared to the s1582 group (Figure 6I‐L). qPCR (Figure 6M) and western blotting (Figure 6N, O) results showed a significant increase in SHANK1 levels in the s1582 +S133D group compared to the s1582 group alone. In addition, s7858 fails to increase SHANK1 levels in neurons transfected with AAV/CREB S133A (Figure S10A, B, Supporting Information). These data further suggest that CREB is required for PKA regulation of SHANK1 expression and neuronal dendritic growth. In addition, Rats’ primary hippocampal neurons were exposed to various concentrations (0.92%,0.95%) of NaCl for 0.5 h at 37°C to simulate a high‐salt environment in vitro (Figure S11A, Supporting Information), in the 0.92% and 0.95% NaCl simulated high salt models, neuronal dendrite loss(Figure S11B, Supporting Information), CREB site 133 dephosphorylation and reduced SHANK1 levels were observed (Figure S11C, D, Supporting Information). However, transfection of the mutant virus CREB‐S133D under 0.92% NaCl conditions alleviated neuronal damage(Figure S11E,F, Supporting Information) and increased SHANK1 levels(Figure S11G,H, Supporting Information). So, these results suggest that CREB‐S133D could rescue the HS‐induced SHANK1 deficiency and neuronal damage.

## 3 Discussion

Globally, dementia affects 6 to 50 million people, with recent publications reporting higher estimates, likely due to increased prevalence or improved diagnosis.^[^ ^42^ ^]^ Neurocognitive disorders affect tens of millions worldwide, imposing a significant societal burden.^[^ ^43^, ^44^ ^]^ However, there are no effective clinical drugs or interventions due to the complex etiology of cognitive dysfunction. Long‐term HS diet is a risk factor for cognitive deficits, but its pathogenesis remains unclear. In our study, we identified that high salt intake in rats leads to the abnormal downregulation of SHANK1 protein. Our findings show that PKA inactivation in HS rats leads to CREB dephosphorylation, contributing to SHANK1 reduction. CREB dephosphorylation by a PKA inhibitor led to decreased SHANK1 levels, dendritic damage, and subsequent cognitive impairments. Notably, PKA activation restored CREB phosphorylation and SHANK1 expression, significantly reversing synaptic and cognitive impairments induced by long‐term HS diet. These results suggest that SHANK1 plays a key role in the pathological and behavioral changes associated with HS diet and indicate SHANK1 as a potential therapeutic target for HS‐related cognitive deficits.

To identify key driving factors of HS‐mediated cognitive dysfunction, we identified SHANK1 as a critical molecule. SHANK is a scaffold protein located in excitatory synapses and is essential for the normal development and function of synapses.^[^ ^21^, ^45^ ^]^ The SHANK protein family, encoded by the SHANK1, SHANK2, and SHANK3 genes, undergoes alternative promoter usage and splicing, generating multiple mRNA transcripts and protein isoforms.^[^ ^20^ ^]^ Although *SHANK* gene mutations have been extensively linked to autism spectrum disorders, the physiological role of SHANK proteins in other contexts, such as cognitive decline, remains underexplored. In the current study, SHANK1 not SHANK2 and SHANK3, was significantly decreased in long‐term HS rats. Our findings enhance the understanding of SHANK1 in synaptic function and provide new insights into how its dysregulation contributes to cognitive impairments associated with long‐term high‐salt diet. This suggests SHANK1's pivotal role in the synaptic and cognitive abnormalities observed in HS model.

Recent findings by Facaro et al. reported that tau phosphorylation in the hippocampus was not evident until 12 weeks. Our studies demonstrated significant cognitive deficits following 9 weeks of chronic HS intake, yet these were not associated with tau phosphorylation, Aβ deposition, or neuronal loss in the hippocampus. Anti‐amyloid therapies have shown modest disease‐modifying benefits. Recent anti‐amyloid monoclonal antibodies have shown modest disease‐modifying effects in early Alzheimer's disease, reducing amyloid‐β plaques and slowing cognitive decline in clinical trials.^[^ ^46^, ^47^, ^48^ ^]^

Given that dementia, including Alzheimer's disease, is characterized by synaptic dysfunction, formation of senile plaques, hyperphosphorylated tau tangles, neuroinflammation, and apoptotic cell death. This suggests that synaptic dysfunction is preexisting in Aβ aggregation and tau pathology and may play a key role in HS diet‐induced cognitive impairments. Our study in 8% HSD‐fed SD rats reveals early dendritic damage occurring before AD‐like pathology, suggesting that this model captures a preclinical window of vulnerability. Importantly, we propose that timely intervention targeting the PKA‐CREB‐SHANK1 axis during this early phase could prevent subsequent AD pathogenesis. While our current data demonstrate HS‐induced synaptic damage and cognitive impairment without tau hyperphosphorylation or Aβ accumulation.

Our findings indicate that HS diet reduces SHANK1 via downregulation of the PKA/CREB pathway, leading to synaptic dysfunction and cognitive deficits in rats, we believe that reducing dietary intake of salt for an extended period of time improves dendritic complexity resulting in a return of cognitive improvements. In addition, we advocate for a low‐salt diet to reduce the risk of developing dementia. However, the PKA/CREB/SHANK1 pathway serves as a potential therapeutic target for cognitive dysfunction in individuals with a high‐salt diet.

## 4 Limitation

In the present study, a limitation of our study was that a long‐term HS diet reduced cAMP levels in the hippocampus, which we do not fully understand the mechanism of this part. Recent studies have found whether GPER1 activation improves hypertension and kidney damage in female Dahl salt‐sensitive rats fed a high salt diet.^[^ ^49^ ^]^ High salt conditions may indirectly modulate cAMP levels in neurons via several GPCR‐dependent mechanisms. First, HS could alter the conformational stability or ligand‐binding affinity of certain GPCRs, especially those sensitive to ionic strength, potentially disrupting their coupling to Gαs/Gαi proteins and subsequent cAMP production.^[^ ^50^, ^51^ ^]^ Second, HS might interfere with the heparan sulfate proteoglycan‐dependent clustering of GPCRs on synaptic membranes, impairing localized cAMP signaling microdomains.^[^ ^52^ ^]^ Third, the elevated Na^+^ concentrations under HS could perturb Na^+^‐sensitive adenylate cyclase and phosphodiesterase, indirectly dysregulating cAMP turnover.^[^ ^53^ ^]^ Notably, SHANK1 downregulation, observed in our HS model, might exacerbate these effects by destabilizing GPCR‐scaffolding complexes, further uncoupling receptors from downstream cAMP effectors.^[^ ^20^, ^54^ ^]^ However, how SHANK1 deficiency disrupts synaptic structure is another limitation. Lack of SHANK1 impairs actin‐cytoskeleton interactions (via βPIX), compromises trans‐synaptic adhesion (through the neuroligin‐GKAP complex), and displaces key glutamate receptors along with their downstream signaling effectors.^[^ ^55^, ^56^ ^]^ These disruptions collectively contribute to the observed dendritic simplification and spine loss, while also potentially exacerbating HS‐induced synaptic dysfunction through impaired GPCR scaffolding.^[^ ^20^ ^]^ In the current study, we adopted an 8% high‐salt diet, following the methodology established by Faraco et al,^[^ ^10^ ^]^ to reliably induce neurovascular and cognitive dysfunction within a controlled experimental timeframe. However, a moderate level of sodium intake over a longer duration would better represent the slow, progressive nature of cognitive decline more typically observed in dementia patients. Therefore, a 2%–4% high‐salt dietary intervention merits additional study to better approximate population‐level sodium exposure.

## 5 Conclusion 

**Figure 7:**
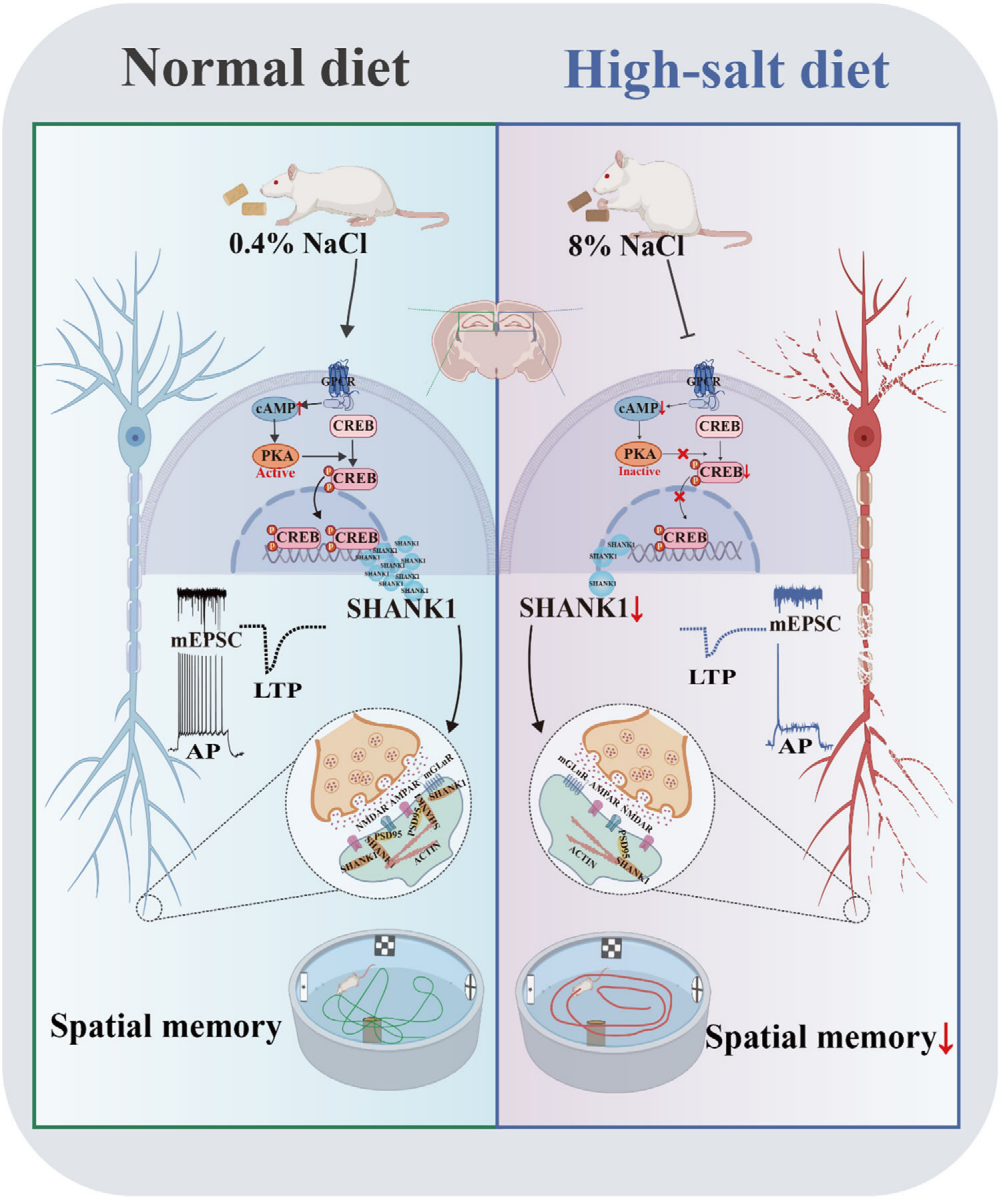
Schematic illustration of the PKA/CREB/SHANK1 axis and its role in synaptic damage and cognitive impairments in high‐salt diet rats. The HS diet downregulates PKA activity, triggering a reduction in CREB phosphorylation. This leads to decreased translocation of the CREB complex into the nucleus and diminished binding to the transcription factor of SHANK1, ultimately resulting in synaptic damage and cognitive impairments.

Our study identifies a novel mechanistic link between long‐term HS diet and cognitive impairment, wherein PKA/CREB axis inactivation leads to SHANK1 reduction, synaptic damage, and cognitive deficits (**Figure** **7**). Given the detrimental effects of PKA/CREB/SHANK1 signaling inhibition on synaptic and cognitive functions in the context of HS diet, targeting the upregulation of this axis may offer a promising therapeutic strategy for addressing HS‐induced cognitive dysfunction.

## 6 Experimental Section

### Reagents

Antibodies and Chemicals used in this study were provided in Supporting Information.

### Animals

Male Sprague‐Dawley (SD) rats were purchased from the Tongji Animal Center at Huazhong University of Science and Technology and allowed to acclimatize in the experimental animal facility for one week. All animal experiments received approval from the Institutional Animal Care and Use Committee (IACUC) of Huazhong University of Science and Technology (IACUC NO.4486).

In our study, 2‐month‐old male SD rats were randomly divided into two groups: one group was fed a normal diet (0.4% NaCl) as a control, while the other group received HS diet (8% NaCl) for 9 weeks. Behavioral tests and electrophysiological and merigenetic tests were used to detect the relevant indicators after 9 weeks (Figure 1A). To further investigate the role of SHANK1, bilateral intra‐hippocampal CA1 injections of AAV‐Sh‐SHANK1 virus were administered to explore its impact on cognitive function. Behavioral test and electrophysiological and merigenetic tests were used to detect the relevant indicators after 4 weeks (Figure 3A). Additionally, either ddH_2_O or the PKA inhibitor (s1582) was bilaterally injected into the hippocampal CA1 region. in 2‐month‐old male SD rats, behavioral testing commenced 48 h post‐injection, sequentially including open field test (day 1), novel object recognition (days 2–3), and fear conditioning (days 4–5). Rats were euthanized following the final behavioral test for subsequent molecular analyses.

For further evaluation of PKA's role in HS‐related cognitive deficits, rats were divided into five groups: a control group on a normal diet (0.4% NaCl), a second group on a HS diet (8% NaCl) for 9 weeks, and other experimental groups on HS diet (8% NaCl) for 9 weeks with bilateral intra‐hippocampal CA1 injections of either ddH_2_O or PKA agonist (2 µL at concentrations of 10, 20, and 30 µm). Behavioral test and electrophysiological and merigenetic tests were used to detect the relevant indicators after 48 h (Figure 5A).

### Feed Preparation

The feed was prepared by Beijing HFK BIOSCIENCE CO., LTD. Feed Main Ingredients: Corn, soybean meal, wheat bran, wheat middlings, fish meal, soybean oil, stone powder, calcium hydrogen phosphate, salt, choline chloride, lysine, multiple vitamins, and multiple mineral elements.

Nutritional Composition: Crude protein 19.2%, crude fat 4.6%, crude fiber 4.0%, crude ash 6.3%, moisture 8.8%, calcium 1.19%, phosphorus 0.87%, lysine 11.1 g kg^−1^, methionine 4.5 g kg^−1^, cystine 6.4 g kg^−1^, nitrogen‐free extract 55.9%. The caloric value was 3.42 kCal g^−1^, with protein accounting for 22.47%, fat for 12.11%, and carbohydrates for 65.42%.

High‐Salt Feed: The salt concentration was increased to 8%, compared to the normal dietary salt concentration of 0.4%. All rats drank tap water normally.

### Stereotactic Surgery

The experimental rats were acclimatized to the operating room one day before surgery. Anesthesia was induced using isoflurane, and the rats were positioned under a stereotactic apparatus. After disinfection with iodophor and 75% ethanol, a midline scalp incision was made between the ears. Bilateral stereotaxic drilling was performed at coordinates 4.2 mm posterior, 3 mm lateral, and 2.8 mm ventral to the bregma. AAV9‐CMV bGlobin‐MCS‐EGFP‐3FLAG‐WPRE‐hGH or Sh‐SHANK1 (2 µL, 2.0 × 10^12 viral genomes per milliliter) was injected into the hippocampal CA1 region at a rate of 0.125 µL/min using a stereotactic apparatus. Similarly, the PKA inhibitor (s1582) or agonist (s7858) was injected into the hippocampal CA1 region. Following the injection, the needle was retained in place for 10 min before being slowly withdrawn. The incision was sutured, and the rats were kept near a heater until they regained consciousness before being returned to the animal room.

### Transmission Electron Microscope

Rats were anesthetized with isoflurane, and tissue from the hippocampal CA1 region was rapidly dissected under a light microscope. Tissues were cut into approximately 10 mm^2^ pieces with a thickness of less than 5 mm. Prior to fixation, samples were rinsed with normal saline or PBS to remove blood and mucus. Tissues from each group were fixed in 2.5% glutaraldehyde at room temperature for 1 h, followed by fixation with 1% OsO_4_ in 0.1M PBS at room temperature for 2 h, then dehydrated and embedded. Images were captured using a light microscope, and semi‐thin slices of 2 µm were prepared and stained with 1% toluidine blue in 1% sodium borate. The CA1 region was identified, and selected semi‐thin sections were further cut into ultra‐thin sections using a Leica microtome, and then stained with uranyl acetate and lead citrate. Ultra‐thin sections were examined using a Hitachi HT7800 transmission electron microscope. The synaptic density, presynaptic vesicles, and the length and width of postsynaptic densities were measured.

### Primary Hippocampal Neuron Culture

Primary hippocampal neurons were isolated from Sprague‐Dawley rat embryos at gestational days 17–18. Following isoflurane anesthesia, the brain was quickly excised and the hippocampus was dissected. The blood vessels on the hippocampal surface were carefully removed using ophthalmic scissors, and the tissue was minced in Hank's buffered saline solution before being suspended in 0.25% (v/v) trypsin solution for 9 min at 37°C. Neurons were plated in 6‐well and 12‐well plates coated with 100 µg mL^−1^ poly‐D‐lysine and incubated at 37°C with 5% CO_2_ for 4 h. The medium was then replaced with neurobasal medium supplemented with 2% (v/v) B‐27 and 1 × GlutaMAX. Half of the neurobasal medium was refreshed every three days. Primary hippocampal neurons were cultured for one week before treatment. Depending on the experimental design, neurons were treated with AAV‐GFP‐Vector/AAV‐Sh‐SHANK1/AAV‐CREB‐S133A/AAV‐CREB‐S133D viruses, PKA inhibitor, PKA agonist, and cycloheximide. Neurons were subsequently collected and lysed in RIPA buffer, or RNA was extracted using Trizol for further bioassays, or they were fixed with 4% paraformaldehyde for immunofluorescence imaging. All cell culture reagents were obtained from Thermo Fisher Technologies. Dendritic complexity was assessed using Sholl analysis, and dendritic length was analyzed using a semi‐automated protocol with Imaris software.

### Western Blotting

Hippocampal tissue was removed from the −80°C freezer and placed on ice. The hippocampal CA1 region or primary hippocampal neurons were isolated and homogenized in a lysis buffer for western blotting. The protein concentration was determined using a bicinchoninic acid (BCA) protein assay kit. Samples were boiled in a water bath for 10 min to promote protein denaturation, then separated on a 10% SDS‐PAGE gel. The running time was adjusted based on the molecular weight of the target protein, and the gel was transferred to a nitrocellulose (NC) membrane. The membrane was incubated in 5% skim milk for 0.5 to 1 h, followed by incubation with the primary antibody at 4°C overnight. The NC membrane was then incubated with IRDye TM (800 CW) anti‐mouse/anti‐rabbit IgG or HRP‐conjugated goat anti‐mouse/anti‐rabbit IgG at 25°C for 1 h, and images were captured using the Odyssey infrared imaging system (LI‐COR Biosciences, USA) or ChemiScope (Clinx Science Instruments Co. Ltd.). Image analysis software was utilized to quantify visual bands.

### Cycloheximide Test

Primary hippocampal neurons were cultured for 7 days. For cycloheximide (CHX) treatment, CHX was diluted in the cell culture medium to a final concentration of 0.1 µm and administered for 0, 3, 6, or 9 h. Following treatment, cells were collected and lysed in RIPA buffer, and protein levels were measured via western blotting.

### Cell Viability Analysis

The survival rate of primary hippocampal neurons was assessed following the manufacturer's instructions using the Cell Counting Kit‐8 (CCK‐8). The cells were seeded in 96‐well plates and treated with different concentrations of the PKA inhibitor s1582 (50, and 100 µm) for 6, 12, and 24 h. A total of 10 µL of CCK‐8 solution was added to 90 µL of medium. After incubation for 2 h at 37°C and 5% CO_2_, absorbance was measured at 450 nm using a microplate reader (BioTek, China).

### Real‐Time Quantitative PCR

Total RNA was extracted using Trizol reagent (Invitrogen, Carlsbad, CA, USA) according to the manufacturer's instructions and reverse‐transcribed into cDNA using a reverse transcription kit (Takara, Dalian, China). A total of 100 ng of cDNA was utilized for real‐time PCR. The reaction mixture was composed as follows: 2 × SYBR Green Mix (5 µL), cDNA (100 ng, 1 µL), forward primer (1µL), reverse primer (1µL), and ddH_2_O (2 µL). The primers for the target genes were designed as follows:

- SHANK1:

Sense: CCTGCGTTCCAAGTCTATGA

Antisense: TTGTTGAGTGCCATCTGATA

- SHANK2:

Sense: CAGCCGCTATTGCCTACC

Antisense: TCTTGTCGTCAGCCCTCC

- SHANK3:

Sense: ACAGGACTACCCGCCTAACC

Antisense: CCGCCATTGCGAAGAAC

- GAPDH:

Sense: TGCCTTCTCTTGTGACAAAGTGG

Antisense: CATTGCTGACAATCTTGAGGGAG

The PCR cycling conditions were set as follows: 95°C for 30 s, followed by 40 cycles of 95°C for 5 s, 60°C for 30 s, and 72°C for 30 s. Melting curve analysis was performed after each experiment. Amplification and analysis were conducted using the StepOnePlus real‐time PCR detection system (Life Technologies, NY, USA).

### Chromatin Immunoprecipitation Quantitative PCR (ChIP‐qPCR)

ChIP was performed using the Simple ChIP® Plus Sonication Chromatin IP Kit (CST, #56383). Fresh or frozen tissue samples (100 mg) were weighed on ice, cut into 1–2 mm pieces, and 1 mL of pre‐cooled PBS with protease inhibitor cocktail (PIC) was added to each sample. Subsequently, 28 µL of 37% formaldehyde solution was added for crosslinking at room temperature for 20 min. Crosslinking was terminated by adding 100 µL of 10X glycine solution, gently mixing, and incubating on ice for 5 min. Nuclear lysis buffer (1 mL) was prepared for chromatin processing and fragmentation. Samples were centrifuged at 21 000 g for 10 min at 4°C, and the supernatant containing cross‐linked chromatin was transferred to a new tube. Chromatin immunoprecipitation (IP) was conducted, and chromatin was eluted from the antibody/protein G microbeads before decrosslinking. To enhance DNA purity, it was purified using a spin column, and ChIP results were quantitatively analyzed by PCR. For SHANK1, an upstream non‐coding sequence of 2000 bp was designed using Gene Software, and divided into 11 segments for RT‐qPCR analysis. Further details can be found in Supporting Information.

### Dual Luciferase Gene Reporter Assay

HEK293 cells were retrieved from liquid nitrogen, quickly thawed in a 37°C water bath, and centrifuged at 1300 rpm for 3 min. The cell suspension was inoculated into a 6 cm dish containing 2 mL of complete medium, gently shaken, and placed in a 37°C, 5% CO_2_ incubator. Cells in the logarithmic growth phase were prepared as suspensions and inoculated into 24‐well plates (approximately 10^5 cells per well, depending on cell morphology), cultured until reaching approximately 60% confluence. Plasmid (1 µg) and Lipo2000 (2 µL) were dissolved in 100 µL of Opti‐MEM per well, mixed, and incubated at room temperature for 20 min before being added to the cells. Luciferase activity was measured 48 h later using a luciferase kit (E1910). For detailed protocols, refer to the Dual‐Luciferase Reporter Assay System Description: Promega Technical Manual. The luciferase plasmid used was SHANK1 (Rat 78957, NM031751 promoter). Vector information included: MCS‐SV40‐firefly Luciferase‐PolyA‐Tk‐Renilla Luciferase‐PolyA.

### cAMP Assay

cAMP levels were measured using an ELISA kit according to the manufacturer's instructions. Freshly collected hippocampal CA1 tissues were immediately frozen and thawed, then tissues were homogenized on ice with a homogenizer, followed by centrifugation at room temperature at 12 000 rpm for 5 min. Experimental procedures included adding 50 µL of Neutralizing Buffer to each well, followed by 50 µL of 0.1 M HCl to the NSB well (Non‐Specific Binding), and adding 50 µL of standard solution at each concentration to corresponding wells. Sample solution (50 µL) was added to corresponding wells, followed by the Assay Buffer working solution and cAMP‐HRP Conjugate solution (50 µL each). Anti‐cAMP monoclonal antibody (50 µL) was then added to each well. Following a 10‐minute incubation at room temperature with TMB Substrate (150 µL), the reaction was stopped with Stop Solution (50 µL), and the optical density was measured at 450 nm using a plate reader to calculate cAMP levels.

### PKA Kinase Activity Assay

PKA activity was measured using the PKA kinase activity kit, a non‐radioactive assay designed for safe, rapid, and reliable measurements. The positive control (active PKA) was prepared, and samples were diluted in Kinase Dilution Buffer in wells. ATP was then added to initiate the kinase reaction, which was incubated for 90 min at 30°C. Phospho‐specific antibody was added to each well and incubated for 60 min at room temperature. After adding the HRP‐conjugated secondary antibody and incubating for another 30 min at room temperature, the wells were washed. TMB solution was added and incubated for 40 min at room temperature, followed by Stop Solution. Optical density was measured at 450 nm, and PKA activity was calculated.

### Fluorescence Imaging and Confocal Microscopy

Brain slices or primary hippocampal neurons were fixed in 4% paraformaldehyde and permeabilized at room temperature in PBS containing 0.5% Triton X‐100 for 30 min. Samples were incubated for 60 min in a 5% BSA solution, followed by overnight incubation at 4°C with target primary antibodies. Afterward, samples were incubated at 37°C for 1 h with Invitrogen fluorescent secondary antibodies (Alexa Fluor 488 or 594), and nuclei were stained with DAPI (0.1 µg/mL). Fluorescence images were captured using a Zeiss LSM 710 laser‐scanning confocal microscope (Zeiss, Jena, Germany) equipped with Zen software. Sholl analysis was conducted to measure dendritic complexity, and dendritic length was assessed using Imaris software (Media Cybernetics, California, USA).

### Golgi Staining

The Golgi solution was prepared as follows: Solution A: 5% potassium dichromate (10 g dissolved in 200 mL ddH₂O); Solution B: 5% mercury chloride (10 g in 200 mL ddH₂O); Solution C: 5% potassium chromate (8 g dissolved in 160 mL ddH₂O). Solutions A and B were mixed to form solution AB; this was then mixed with 400 mL ddH₂O containing solution C. The final volume ratio of potassium dichromate: mercury chloride: potassium chromate: water was 5:5:4:10. The solution was stored in the dark for approximately one week. Sampling involved anesthetizing rats with isoflurane, injecting approximately 300 mL of normal saline into the left ventricle to clear the brain of invading vesicles, and immersing brain tissues in dark AC solution for 30 days, changing the Golgi solution every 2 days. Brain tissue was sectioned using a vibrating microtome (Leica, Wetzlar, Germany) at a thickness of 100 µm and dried on slides at room temperature. Staining involved alkalizing samples with ammonia, dehydrating, and soaking in CXA solution for 15 min (1:1:1 mixture of 1000 mL chloroform, 1000 mL xylene, and 1000 mL anhydrous ethanol), then sealing with neutral gum and allowing to dry. Image‐Pro Plus software was used for image analysis.^[^ ^57^ ^]^

### Acute Slice Preparation and LTP Recording

Rats were decapitated under isoflurane anesthesia, and brains were rapidly excised and placed in ice‐cold, oxygenated artificial cerebrospinal fluid (aCSF) composed of: 119 mm NaCl, 2.5 mm KCl, 26.2 mm NaHCO₃, 1 mm NaH₂PO₄, 11 mm glucose, 1.3 mm MgSO₄, and 2.5 mm CaCl₂ (pH 7.4). Coronal brain slices of 350 µm thickness were prepared using a vibratome (Leica, VT1000S, Germany) and subsequently transferred to a recovery chamber filled with oxygenated aCSF for a minimum of 1.5 h at room temperature prior to electrophysiological recordings. For long‐term potentiation recordings, acute brain slices were submerged in aCSF within a recording chamber. The slices were positioned on an 8 × 8 microelectrode array (Parker Technology, Beijing, China), featuring 50 × 50 µm electrodes with a 150 µm interelectrode distance. Electrical signals were recorded using the MED64 system (Alpha MED Sciences, Panasonic), targeting field excitatory postsynaptic potentials (fEPSP) in CA1 neurons by stimulating CA3 neurons. LTP induction was accomplished via three trains of high‐frequency stimulation (HFS; 100 Hz, 1 s duration). The magnitude of LTP was quantified as the percentage change in the fEPSP slope, measured between 60–90 min post‐induction.^[^ ^57^, ^58^, ^59^ ^]^

### Recording of mEPSC

Neurons in the CA1 hippocampal slice were visualized using a DIC‐infrared upright microscope and recorded using whole‐cell patch clamp procedures at ‐70 mV. The pipette resistance was in the range of 4–6MΩ. The intracellular solution contained (in mm): 140 K‐gluconate, 2 MgCl_2_, 10 HEPES, 10 BAPTA, 2 Mg‐ATP, 0.5 CaCl_2_‐H_2_O, 0.5 Li‐GTP, pH 7.2‐7.4, 280–290 mOsm. 5mm QX‐314 was added to block voltage‐gated Na+ channels and GABAB receptors. The mEPSCs were recorded in the presence of 1 µm tetrodotoxin (TTX), and 100 µm picrotoxin (PTX).

### Recording of mIPSCs

The pipette electrodes, with a resistance of 3–5MΩ, were filled with the internal solution containing (in mm): 140 CsCl, 10 HEPES, 2 MgCl_2_, 0.5 EGTA, 2 MgATP, 0.5 Na_3_GTP, 12 phosphocreatine and 30 NMG, pH 7.2‐7.4, 280–290 mOsm. 5mm QX‐314 was added to block voltage‐gated Na+ channels and GABABRs. After the neuron was voltage‐clamped at −70 mV, mIPSCs were recorded in the presence of 5 µm TTX and 50 µM kynurenic acid (KYN).

### Recording of Paired Pulse Ratio

Paired pulse ratios were calculated as a ratio of EPSC2 to EPSC1 separated by inter‐stimulus intervals of 50, 100, 150, and 200 ms for EPSCs. To measure the input‐output curves for pyramidal neurons, a bipolar tungsten electrode was placed in the stratum radiatum with a bipolar tungsten electrode ≈50 um from the recording electrode in order to test potential presynaptic effects.

### Recording of AP

Recordings were obtained using borosilicate glass micropipettes (3–8 MΩ). The internal pipette solution consisted of the following (in mm): 145 K‐gluconate, 10 HEPES, 1.0 EGTA, 2.0 Na_2_ATP, and 0.4 NaGTP (300 mOsmol/kgH_2_O, pH 7.2). Neurons were slightly depolarized with current injection (500 ms, current clamp) to generate a regular spiking activity (range, ‐50 to 230 pA).

### Behavior Test–Open Field Test

The open field test was employed to assess anxiety and exploratory behaviors. The apparatus consisted of a 100 × 100 cm^2^ PVC square arena with 70 cm high walls. Individual rats were placed in the arena for a 5 min session. Anxiety levels were quantified by the number of entries into the center of the arena, while exploratory activity was measured by tracking the total distance covered by the rats. Data collection utilized tracking files recorded with LabState version 1.0 software (AniLab Software and Instruments Co., LTD., China).

### Elevated Plus Maze

This test was designed to evaluate the conflict between exploration of a new environment and fear of the elevated open arms, reflecting anxiety in rats. The elevated plus maze featured two open arms (50 cm long × 10 cm wide), two closed arms (50 cm long × 10 cm wide × 40 cm high), and a central platform (10 cm × 10 cm), elevated 70 cm above the ground. Rats were acclimated in the testing room for 24 h prior to the experiment. During the test, each rat was positioned in the maze's center and observed for 5 min, The tines and duration of entries into the open and closed arms were recorded. The open arm duration ratio = Duration in open arm / (Duration in open arm + Duration in closed arm) and the open arm entry ratio where open arm entry ratio = (open arm entries) / (open arm entries + closed arm entries).

### Social Interaction Test

The experimental setup consisted of three boxes, each measuring 19 cm × 45 cm, separated by transparent plexiglass with a central channel for connectivity. A uniform metal cage, sized appropriately for a rat, was placed centrally in the left and right boxes. Prior to testing, rats were acclimated in the behavioral testing room for 30 min. A novel rat was positioned in the metal cage on one side, while the opposite cage remained empty. Initially, the three boxes were separated by a transparent glass resin plate, and the test rat was placed in the third box for 5 min. Following this, the glass resin plate was removed, permitting the test rat to explore the three boxes freely for an additional 5 min. The time spent interacting with familiar and unfamiliar rats was meticulously recorded using AniLab Software, which tracked and quantified interaction durations based on predefined proximity criteria. The track plots were generated by the software to visualize these interactions.

### Novel Objective Recognition Test

Rats were placed in the novel object recognition room 24 h prior to the test and subsequently transferred to a 100 cm × 100 cm × 100 cm plastic container for a 5 min familiarization period with objects A and B. After each familiarization phase, the arena and objects were cleaned with 70% ethanol. 2 h following the initial familiarization, object B was replaced with object C, and rats were allowed 5 min to explore both objects. After 24 h, object C was swapped for object D, and rats were again given 5 min to explore. The duration of exploration for objects C and D was recorded during both the 2 and 24 h intervals. The recognition index (RI) was calculated as: RI (2h) = time at object C [(time at object C) / (time at objects A and C)], RI(24h) = time at object D [(time at object D)/(time at objects A and D)].

### Morris Water Maze Test

The Morris water maze was utilized to assess spatial learning and memory. Rats were acclimated to the water maze room for 24 h, where the walls were adorned with distinct patterns to facilitate environmental orientation. During the training phase, rats were tasked with locating a hidden platform over five to six consecutive days, with four trials conducted each day at intervals of 40 min between 8:00 AM and 2:00 PM. Each trial commenced in one of four quadrants, with the rat facing the pool wall, concluding when the rat successfully climbed onto the platform. If a rat fails to locate the platform within 1 minute, the experimenter would manually guide it to the platform, where it remained for 20 s. A camera recorded the swim paths and time taken to find the platform. Spatial memory was evaluated following the training phase; on day 6 or 7, the platform was removed, and the duration spent in the target quadrant and the number of platform crossings was recorded.

### Fear Condition

Fear conditioning was employed to evaluate the effect of contextual training on memory consolidation. On the initial day, rats were acclimated to the conditioning chamber for a duration of 3 min, after which an auditory stimulus (70 dB, 30 s) was presented, followed by a foot shock (0.5 mA, 2 s). This sequence was replicated twice, with a 2‐minute interval between each trial. Two h post‐conditioning, the rats were reintroduced to the chamber for 3 min in the absence of any stimuli, during which freezing behavior was recorded as a measure of memory retention. The following day, rats were placed back in the chamber for an additional 3 min without any stimuli to evaluate contextual recall by quantifying the duration of freezing behavior.

### RNA Sequencing

Tissue samples were obtained from the rat hippocampal CA1 region. Following appropriate pretreatment, samples were sent to Novogene Sequencing Company (Tianjin, China) for sequencing. RNA was extracted from tissues using standard extraction methods, followed by stringent quality control to ensure an accurate assessment of RNA integrity. After library construction, initial quantification was performed using a Qubit 2.0 Fluorometer, and the library was diluted to 1.5 ng µL^−1^. The effective concentration of the library was accurately quantified using qRT‐PCR to ensure quality. Upon qualification of the library test, Illumina sequencing was conducted following the pooling of different libraries.

### Data Sets Collection and Processing

In this study, RNA‐seq was conducted to identify marker genes associated with HS diet rats in the hippocampus. The datasets GSE36980^[^ ^60^ ^]^ and GSE84422^[^ ^61^ ^]^ were obtained from the Gene Expression Omnibus (GEO) database, and corresponding mRNA expression matrices were aligned for comprehensive analysis. In the GSE36980 dataset, analysis was performed on postmortem human brain tissues donated for the Hisayama study to identify molecular pathological changes in AD brains. The control group consisted of hippocampal samples GSM907861‐GSM907870, which served as references for bioinformatics analysis. The experimental group included hippocampal samples from AD patients (GSM907854‐GSM907860). mRNA expression matrices were normalized using the “limma” R package.

### Bioinformatics Analysis

Differential Expression Genes (DEGs) were identified using the “DESeq2” R package, with the significance threshold set at p‐value < 0.05. A volcano plot was generated using the “EnhancedVolcano” R package to visualize the DEG results. GO and KEGG pathway enrichment analyses were performed using the “biomaRt” and “clusterProfiler” R packages, with a p‐value < 0.05 considered statistically significant. The results of KEGG and GO enrichment analyses were visualized using the “ggsankey”, “GOplot”, and “ggplot2” R packages. A total of 14 co‐upregulated and 40 co‐downregulated genes were identified from the HS diet models and the GSE36980 dataset. Venn diagrams were used to illustrate the intersection of these 54 co‐DEGs with 12 core genes involved in the synapse‐related pathway including the “Synaptic Vesicle Cycle”, “Glutamatergic Synapse”, and “GABAergic Synapse” pathways identified through KEGG pathway enrichment analysis. Protein‐Protein Interaction (PPI) networks of the 12 core genes were constructed using the brain network (with an edge weight > 0.5) from the TissueNexus online database to explore their interactions. Pearson correlation analysis was conducted using the “WGCNA”, “ggpmisc” and “ggpubr” R packages to investigate the relationships between the key genes with clinical features.

### Statistical Analysis

Data were expressed as mean ± SD and were analyzed using GraphPad Prism 8.0 statistical software (GraphPad Software, https://www.graphpad.com/scientific‐software/prism/). A two‐tailed *t*‐test was utilized to assess variance between two groups, while differences among multiple groups were determined using one‐ or two‐way analysis of variance (ANOVA) followed by Tukey's multiple comparisons test or Sidak's multiple comparisons test. A *p*‐value < 0.05 was deemed statistically significant.

## Conflict of Interest

The authors declare no conflict of interest.

## Author Contributions

X.W. designed, planned, and organized all experiments and results, including the writing of the manuscript. C.G. and W.L. planned and performed all experiments. H.S., Y.L., and T.W. guided and executed electrophysiology. Y.L., Y.A.R.M., and W.L. analyzed and interpreted the data. J.W., R.L., and Y.G. proofread our manuscript. All authors read and approved the final manuscript.

## Ethics Approval and Consent to Participate

No humans were used in this research. All animal experiments were approved by the Animal Care and Use Committee of Huazhong University of Science and Technology and performed in compliance with the National Institutes of Health Guide for the Care and Use of Laboratory Animals.

## Supporting information



Supporting Information

Supporting Information

Supporting Information

## Data Availability

The data that support the findings of this study are available from the corresponding author upon reasonable request.
