# Supplementary material for: Long Term High‐Salt Diet Induces Cognitive Impairments via Down‐Regulating SHANK1
Source: Adv Sci (Weinh). 2025 Jun 26;12(36):e02099. doi: 10.1002/advs.202502099 (PMC12463032; doi:10.1002/advs.202502099)
Supplement: Supplementary file 1 — Supporting Information [file ADVS-12-e02099-s001.docx]

**Statistical analysis for Figure 1-6 and Figure S1-11**

| **Figure** | **Statistic method** | **Number(n)** | **Statistic results** | **Value** |
| --- | --- | --- | --- | --- |
| 1C | Two-tailed  Two-tailed | 19,19  19,19 | P=0.0013  P=0.0053 | t=3.480, df=36  t=2.969, df=36 |
| 1D | Two-tailed | 19,19 | P= 0.8277 | t=0.2193, df=36 |
| 1E | Two-tailed | 19,19 | P= 0.0259 | t=2.323, df=36 |
| 1F | Two-tailed | 19,19 | P=0.0172 | t=2.497, df=36 |
| 1G | Two-tailed | 19,19 | P<<0.0001 | t=4.410, df=36 |
| 1H | Two-tailed | 19,19 | P<<0.0001 | t=5.322, df=36 |
| 1L | Two-tailed | 3,3 | P= 0.0039 | t=5.991, df=4 |
| 1N | Two-tailed | 6,6 | P=0.8951 | t=0.1352, df=10 |
| 1P | Two-tailed  Two-tailed | 11,9  11,9 | P=0.0089  P=0.0328 | t=2.934, df=18  t=2.312, df=18 |
| 1R | Two-tailed  Two-tailed | 12,14  12,14 | P=0.4803  P=0.3939 | t=0.7170, df=24  t=0.8682, df=24 |
| 1T | Two-tailed | 10,10 | P=0.0006 | t=4.163, df=18 |
| 1U | Two-tailed | 48,43 | P=0.0025 | t=3.107, df=89 |
| 1V | Two-tailed | 47,43 | P=0.6806 | t=0.4131, df=88 |
| 1W | Two-tailed | 48,43 | P=0.8033 | t=0.2499, df=89 |
| 2F | Two-tailed  Two-tailed | 3,3  3,3 | P=0.0385  P=0.0174 | t=3.037, df=4  t=3.910, df=4 |
| 2G | Two-tailed  Two-tailed  Two-tailed | 4,4  4,4  4,4 | P=0.0054  P=0.8871  P=0.1851 | t=4.248, df=6  t=0.1481, df=6  t=1.497, df=6 |
| 2I | Two-tailed | 3,3 | P=0.0063 | t=5.260, df=4 |
| 3C | Two-tailed | 3,3 | P=0.0099 | t=4.619, df=4 |
| 3D | Two-tailed | 12,15 | P=0.5390 | t=0.6228, df=25 |
| 3E | Two-way ANOVA with Sidak's multiple comparisons test | 12,15 | P=0.1094  P<0.0001  P<0.0001 | F (5, 150) = 1.834  F (5, 150) = 30.90  F (1, 150) = 21.51 |
| 3F | Two-tailed | 12,15 | P=0.0104 | t=2.769, df=25 |
| 3G | Two-tailed | 12,15 | P=0.0075 | t=2.909, df=25 |
| 3H | Two-tailed | 12,15 | P=0.0025 | t=3.355, df=25 |
| 3I | Two-tailed | 12,15 | P=0.0013 | t=3.614, df=25 |
| 3M | Two-tailed | 3,3 | P= 0.0076 | t=4.978, df=4 |
| 3O | Two-tailed  Two-tailed | 10,14  10,14 | P=0.0005  P=0.0186 | t=4.098, df=22  t=2.541, df=22 |
| 3S | Two-tailed  Two-tailed | 30,30  30,30 | P<0.0001  P<0.0001 | t=4.643, df=58  t=8.687, df=58 |
| 3U | Two-tailed | 9,9 | P=0.0081 | t=3.022, df=16 |
| 3V | Two-tailed | 44,40 | P<0.0001 | t=4.163, df=82 |
| 3W | Two-tailed | 44,40 | P=0.0421 | t=2.065, df=82 |
| 3X | Two-tailed | 44,40 | P=0.2486 | t=1.162, df=82 |
| 4A | Two-tailed | 6,6 | P=0.0009 | t=4.650, df=10 |
| 4B | Two-tailed | 6,6 | P=0.0001 | t=6.108, df=10 |
| 4D | Two-tailed | 3,3 | P=0.0002 | t=13.73, df=4 |
| 4E | One-way ANOVA with Tukey's multiple comparisons test | 3,3,3,3 | P<0.0001 | F (3, 8) = 35.58 |
| 4F | Two-tailed,  Two-tailed | 3,3  3,3 | P=0.0334  P=0.0005 | t=3.186, df=4  t=10.37, df=4 |
| 4G | One-way ANOVA with Tukey's multiple comparisons test | 6,6,6,5 | P<0.0001 | F (3, 19) = 24.70 |
| 4I | One-way ANOVA with Tukey's multiple comparisons test | 3,3,3,3 | P<0.0001 | F (3, 8) = 55.47 |
| 4J | One-way ANOVA with Tukey's multiple comparisons test | 3,3,3,3 | P=0.0007 | F (3, 8) = 17.57 |
| 4K | One-way ANOVA with Tukey's multiple comparisons test | 3,3,3,3 | P<0.0001 | F (3, 8) = 40.25 |
| 4P | One-way ANOVA with Tukey's multiple comparisons test | 15,15,15,15 | P<0.0001 | F (3, 56) = 56.43 |
| 4Q | One-way ANOVA with Tukey's multiple comparisons test | 15,15,15,15 | P<0.0001 | F (3, 56) = 69.37 |
| 4R | One-way ANOVA with Tukey's multiple comparisons test | 7,8,7,7 | P=0.0636 | F (3, 25) = 2.754 |
| 4S | One-way ANOVA with Tukey's multiple comparisons test | 7,8,7,7 | P<0.0001 | F (3, 25) = 11.49 |
| 4T | One-way ANOVA with Tukey's multiple comparisons test | 3,3,3,3  3,3,3,3 | P=0.0003  P=0.0018 | F (3, 8) = 23.01  F (3, 8) = 13.33 |
| 5B | One-way ANOVA with Tukey's multiple comparisons test | 4,4,4,4 | P<0.0001 | F (4, 15) = 13.73 |
| 5C | One-way ANOVA with Tukey's multiple comparisons test | 3,3,3,3 | P<0.0001 | F (4, 10) = 19.94 |
| 5E | One-way ANOVA with Tukey's multiple comparisons test | 3,3,3,3,3 | P<0.0001 | F (4, 10) = 21.77 |
| 5F | One-way ANOVA with Tukey's multiple comparisons test | 3,3,3,3,3 | P<0.0001 | F (4, 10) = 16.99 |
| 5G | One-way ANOVA with Tukey's multiple comparisons test | 10,8,7,7,7 | P=0.8774 | F (4, 34) = 0.2977 |
| 5H | One-way ANOVA with Tukey's multiple comparisons test | 10,8,7,7,7 | P= 0.0002 | F (4, 34) = 7.304 |
| 5I | One-way ANOVA with Tukey's multiple comparisons test | 10,8,7,6,7 | P=0.0043 | F (4, 33) = 4.656 |
| 5J | One-way ANOVA with Tukey's multiple comparisons test | 10,8,7,6,7 | P=0.0013 | F (4, 33) = 5.737 |
| 5L | One-way ANOVA with Tukey's multiple comparisons test | 3,3,3 | P<0.0001 | F (2, 6) = 111.2 |
| 5P | One-way ANOVA with Tukey's multiple comparisons test | 25,25,25 | P<0.0001 | F (2, 72) = 44.76 |
| 5Q | One-way ANOVA with Tukey's multiple comparisons test | 25,25,25 | P<0.0001 | F (2, 72) = 43.23 |
| 6B | One-way ANOVA with Tukey's multiple comparisons test | 3,3,3 | P=0.0002 | F (2, 6) = 52.20 |
| 6D | One-way ANOVA with Tukey's multiple comparisons test | 4,4,4,4 | P<0.0001 | F (2, 9) = 112.2 |
| 6G | One-way ANOVA with Tukey's multiple comparisons test | 15,15,15 | P<0.0001 | F (2, 42) = 28.17 |
| 6H | One-way ANOVA with Tukey's multiple comparisons test | 15,15,15 | P<0.0001 | F (2, 42) = 51.04 |
| 6K | One-way ANOVA with Tukey's multiple comparisons test | 16,16,16 | P=0.0018 | F (2, 45) = 7.283 |
| 6L | One-way ANOVA with Tukey's multiple comparisons test | 15,15,15 | P=0.0001 | F (2, 42) = 11.42 |
| 6M | One-way ANOVA with Tukey's multiple comparisons test | 5,5,5 | P=0.0009 | F (2, 12) = 13.37 |
| 6O | One-way ANOVA with Tukey's multiple comparisons test | 4,4,4 | P<0.0001 | F (2, 9) = 43.16 |
| S1A | Two-tailed | 19,19 | P=0.8594 | t=0.1784, df=36 |
| S1B | Two-tailed | 19,19 | P= 0.8820 | t=0.1494, df=36 |
| S1C | Two-tailed | 19,19 | P=0.0041 | t=3.067, df=36 |
| S1D | Two-tailed | 19,19 | P=0.8339 | t=0.2113, df=36 |
| S1E | Two-tailed | 19,19 | P=0.2034 | t=1.295, df=36 |
| S1F | Two-tailed | 19,19 | P=0.0258 | t=2.325, df=36 |
| S2A | Two-tailed | 6,6 | P= 0.4569 | t=0.7740, df=10 |
| S2B | Two-tailed | 4,6 | P= 0.2967 | t=1.116, df=8 |
| S5A | Two-tailed | 12,15 | P= 0.1605 | t=1.446, df=25 |
| S5B | Two-tailed | 12,15 | P= 0.2718 | t=1.124, df=25 |
| S5C | Two-tailed | 12,15 | P=0.3944 | t=0.8666, df=25 |
| S5D | Two-tailed | 12,15 | P= 0.0240 | t=2.402, df=25 |
| S6C | Two-tailed | 15,15 | P= 0.0004 | t=4.044, df=28 |
| S8A | 2way ANOVA with Tukey's multiple comparisons test | 6,6,6  6,6,6  6,6,6 | P=0.2125  P=0.1825  P=0.3736 | F (4, 45) = 1.520  F (2, 45) = 1.767  F (2, 45) = 1.006 |
| S9A | One-way ANOVA with Tukey's multiple comparisons test | 5,5,5,5 | P=0.0001 | F (3, 16) = 13.93 |
| S9B | One-way ANOVA with Tukey's multiple comparisons test | 7,8,7,7 | P=0.3318 | F (3, 25) = 1.195 |
| S9C | One-way ANOVA with Tukey's multiple comparisons test | 7,8,7,7 | P=0.0250 | F (3, 25) = 3.693 |
| S9D | One-way ANOVA with Tukey's multiple comparisons test | 7,8,7,7 | P=0.1987 | F (3, 25) = 1.671 |
| S9E | One-way ANOVA with Tukey's multiple comparisons test | 7,8,7,7 | P=0.0098 | F (3, 25) = 4.697 |
| S10B | One-way ANOVA with Tukey's multiple comparisons test | 4,4,4 | P<0.0001 | F (2, 9) = 59.58 |
| S11B | One-way ANOVA with Tukey's multiple comparisons test | 10,10,10 | P<0.0001 | F (2, 27) = 50.76 |
| S11D | One-way ANOVA with Tukey's multiple comparisons test | 4,4,4  4,4,4 | P<0.0001  P<0.0001 | F (2, 9) = 78.03  F (2, 9) = 62.28 |
| S11F | One-way ANOVA with Tukey's multiple comparisons test | 10,10,10 | P<0.0001 | F (2, 27) = 27.83 |
| S11H | One-way ANOVA with Tukey's multiple comparisons test | 4,4,5 | P=0.0009 | F (2, 9) = 16.78 |
