# Supplementary material for: Long Term High‐Salt Diet Induces Cognitive Impairments via Down‐Regulating SHANK1
Source: Adv Sci (Weinh). 2025 Jun 26;12(36):e02099. doi: 10.1002/advs.202502099 (PMC12463032; doi:10.1002/advs.202502099)
Supplement: Supplementary file 2 — Supporting Information [file ADVS-12-e02099-s002.docx]

**Supplementary Figure 1**

**
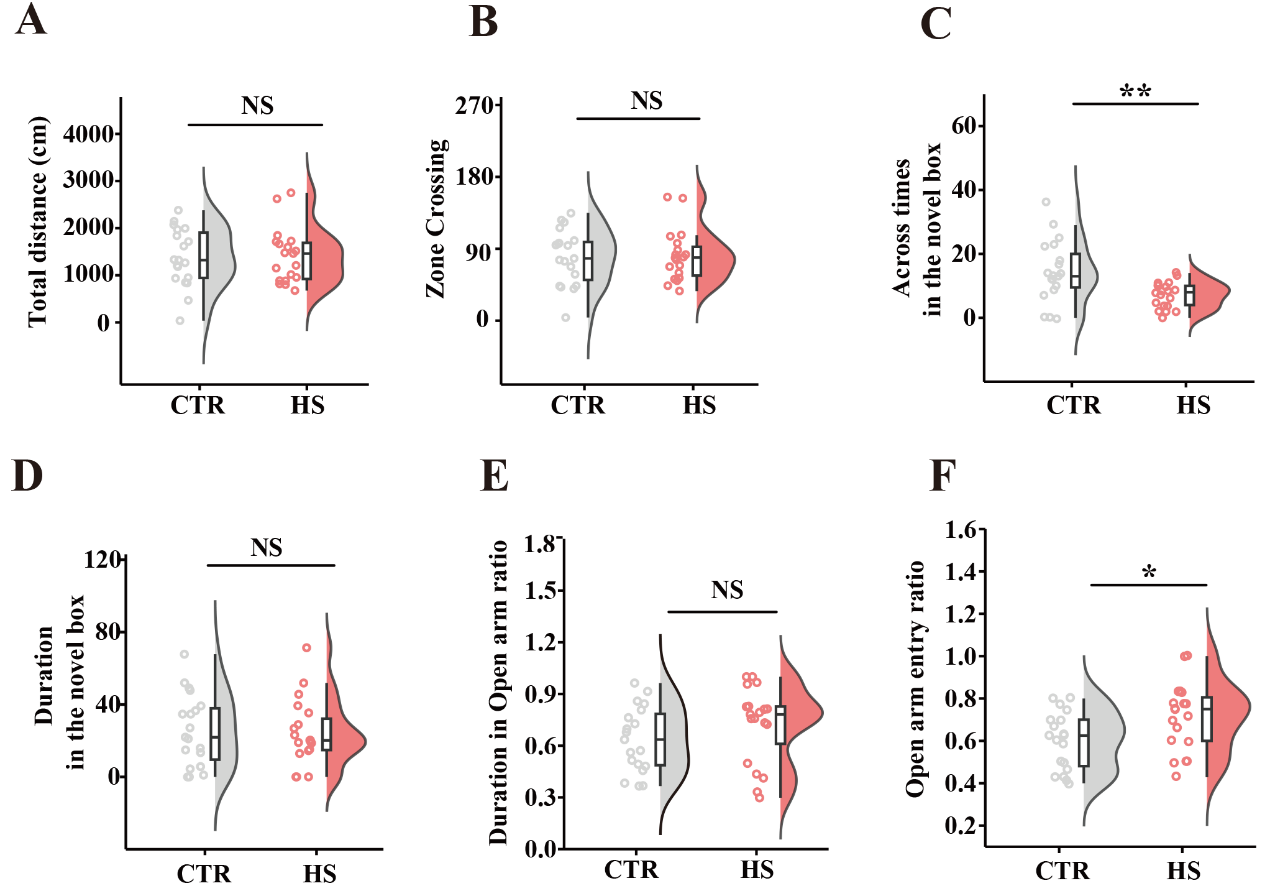
**

**sFigure 1 High-salt diet induces behavioral dysfunction**

The open field test measured the total distance covered (A) and zone crossing (B) in the two groups, (n=19). Three boxes of social experiments showed the social ability of rat, the times (C) and the duration (D) of entering the box of strange rats were measured, (n=19). Elevated plus maze test was measured the duration in Open arm ratio (E) and Open arm entry ratio (F), (n=19). All data represent as mean ± SD. *P < 0.05, **P < 0.01, vs control. Statistical details were provided in Supporting information 1.

**Supplementary Figure 2**

**
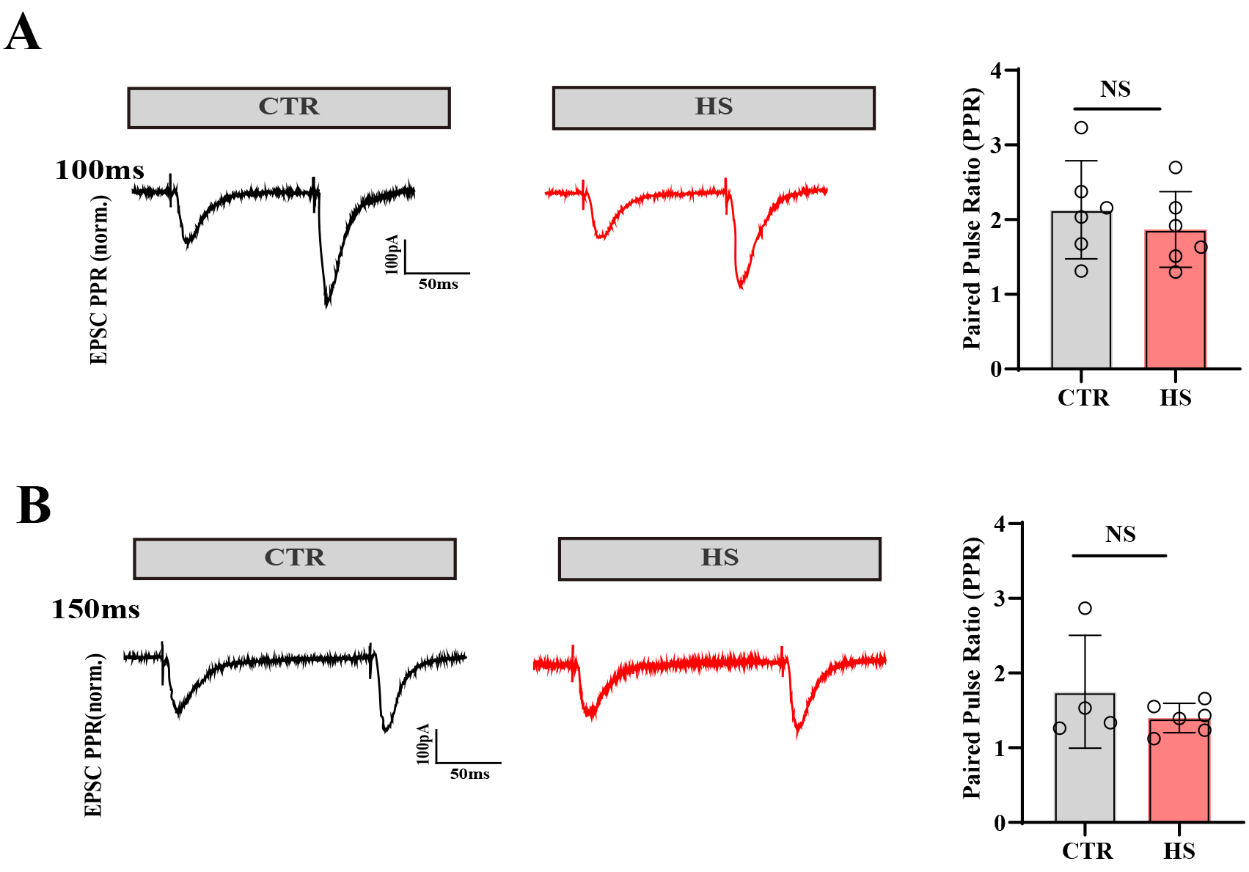
**

**sFigure 2** **PPR results showed no significant difference between the control and HS groups**

For pairwise pulse ratio experiments, paired stimuli 100ms (A), 150ms (B) were delivered. All data represent as mean ± SD. Statistical details were provided in Supporting information 1.

**Supplementary Figure 3**

**
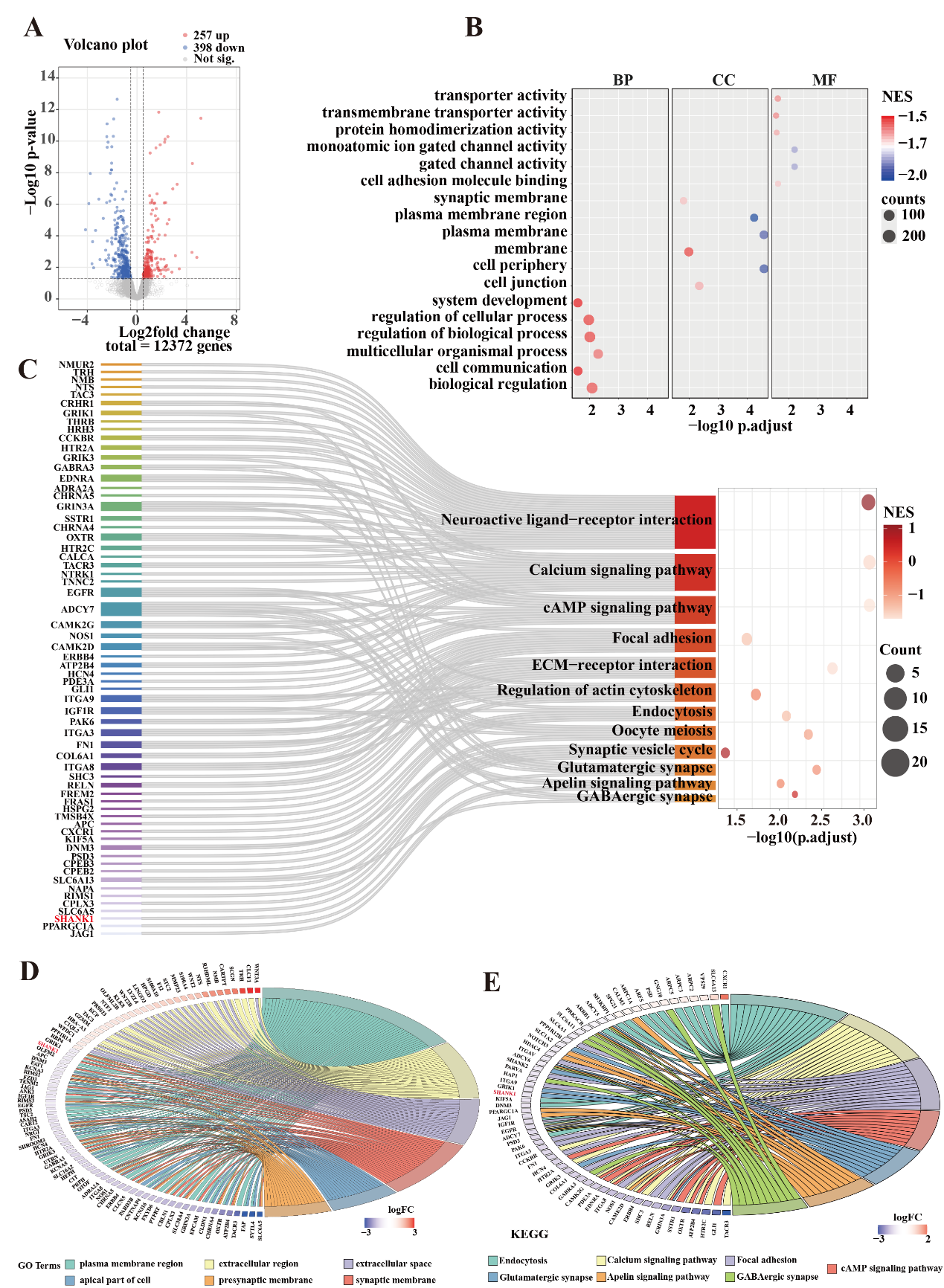
**

**sFigure 3 The visualization of RNA sequencing analysis results**

(A) Volcano plot illustrating the differentially expressed genes from RNA-seq analysis of the high-salt diet rat model. (B) Gene Ontology (GO) enrichment analysis results for DEGs from RNA-seq analysis of the high-salt diet rat model. (C) KEGG pathways enriched in DEGs from RNA-seq analysis of a high-salt diet rat model. (D) Visualization of top six enriched cellular component and the core enrichment genes identified from GO enrichment analysis results. (E) Visualization of top seven KEGG pathways enriched with the core enrichment genes.

**Supplementary Figure 4**

**
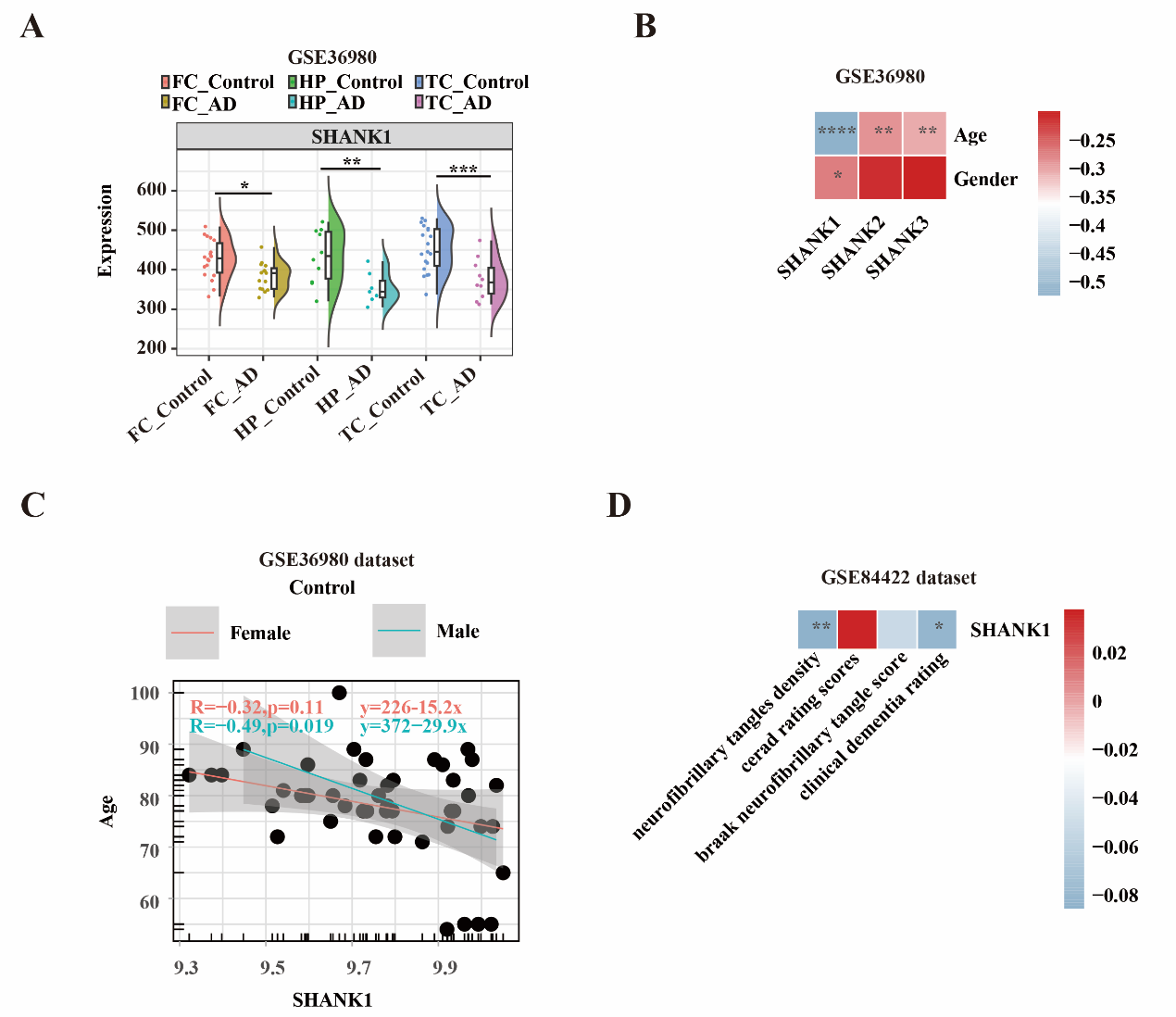
**

**sFigure 4 The visualization of GSE36980 analysis results**

(A) Expression levels of SHANK1 in different regions of AD and non-AD human brains, including the temporal cortex, hippocampus and frontal cortex, based on data from the GSE36980 dataset. (B) Correlation analysis of the expression levels of Shank1, Shank2, and Shank3 with age and gender in the GSE36980 dataset. (C) Visualization of correlation of the expression level of Shank1 with age in non-AD human samples from the GSE36980 dataset, the correlation between SHANK1 expression and age is particularly strong in males (C). Correlation analysis in GSE84422 reveals a negative association between SHANK1 expression and both neurofibrillary tangle density and clinical dementia rating (D). All data represent as Pearson Correlation Analysis. *P < 0.5, **P < 0.01, ***P < 0.001, ****P < 0.0001vs control.

**Supplementary Figure 5**

**
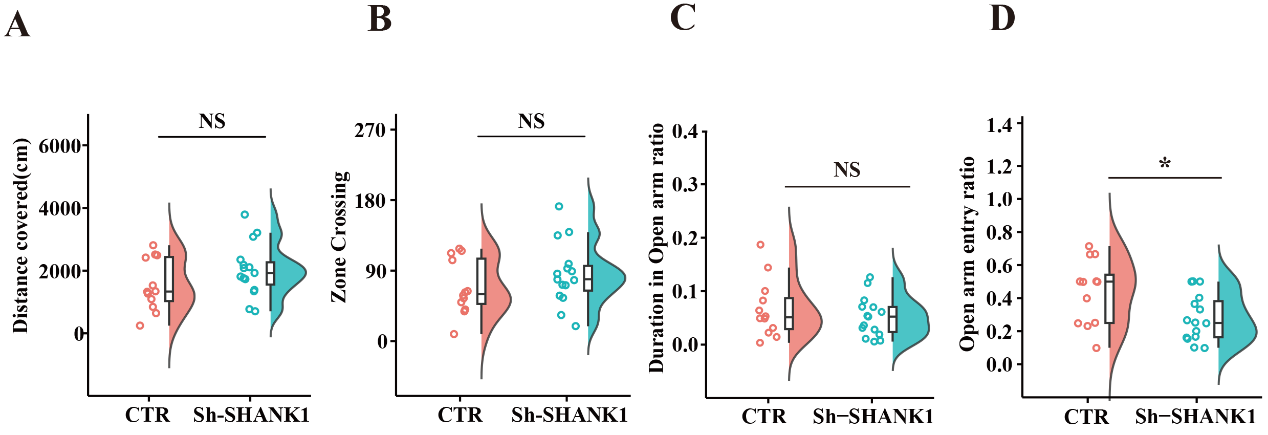
**

**sFigure 5 Downregulation of SHANK1 results in behavior disorder**

(A, B) The open field test measured the total distance covered (A) and zone crossing (B) in the two groups, (n=12-15). (C, D) Elevated plus maze test was measured the duration in Open arm ratio (C) and open arm entry ratio (D), (n=19). All data represent as mean ± SD. *P < 0.5 vs control. Statistical details were provided in Supporting information 1.

**Supplementary Figure 6**

**
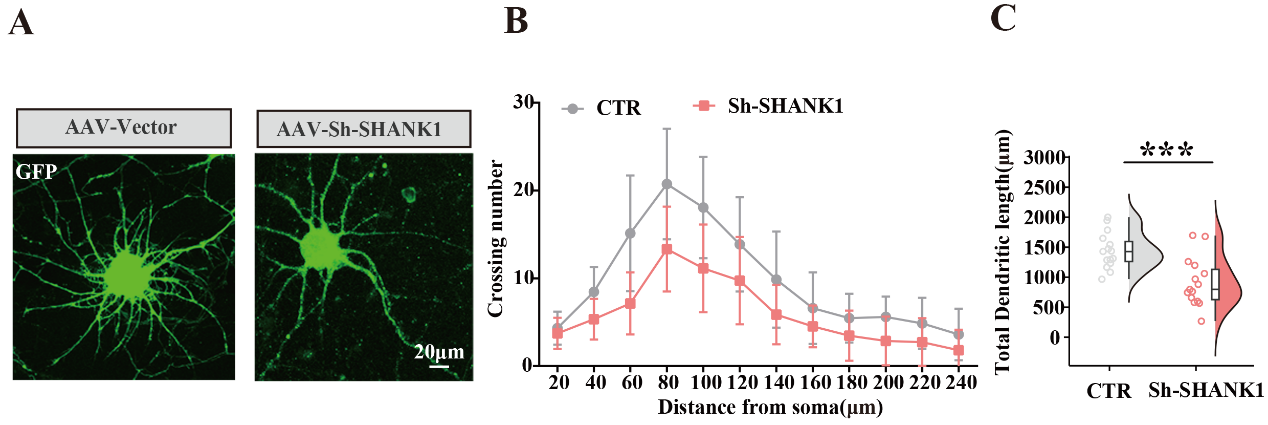
**

**sFigure 6 Downregulation of SHANK1 results in neuronal damage**

(A-C) Rats’ primary hippocampal neurons were treated with AAV-GFP-vector for control and AAV-GFP-Sh-SHANK1 for model for 48 hours, representative images after treatment (A) Scale bar = 20 μm), Sholl analysis (B), quantitative analyses of dendritic length (C), (n = 15 hippocampal neurons). All data represent mean ± SD. ***p < 0.001 versus control. Statistical details were provided in Supporting information 1.

**Supplementary Figure 7**

**
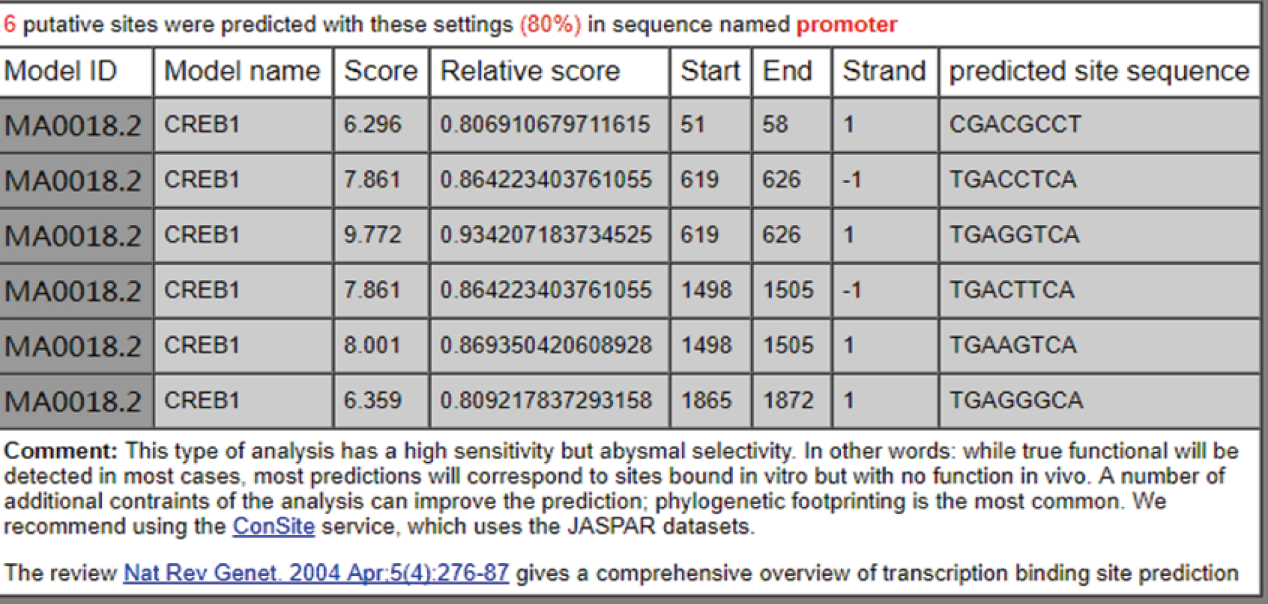
**

**sFigure7 CREB regulates the transcription of SHANK1**

JASPER software predicts that CREB may be a potential transcription factor of SHANK1 gene.

**Supplementary Figure 8**

**
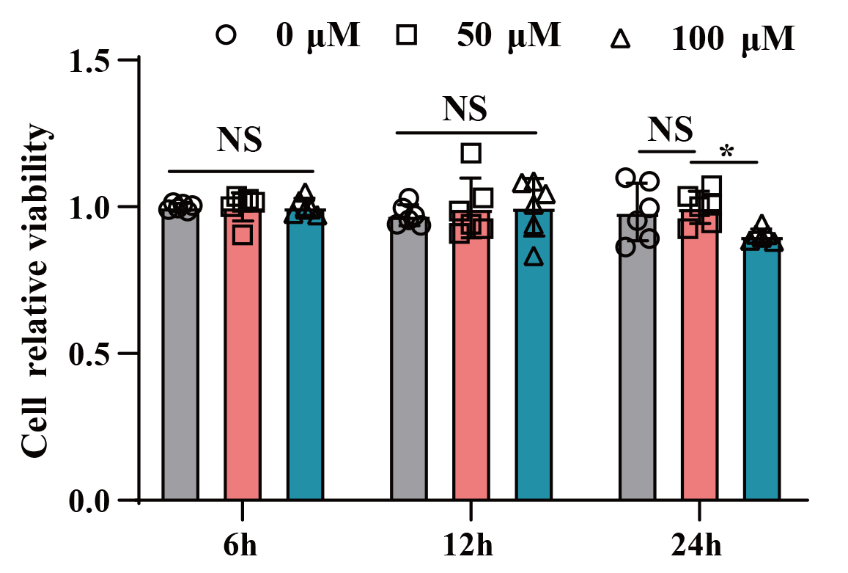
**

**sFigure 8 CCK-8 assay on cytotoxicity of PKA inhibitor s1582 in primary hippocampal neuron**

(A) CCK-8 assay on cytotoxicity of PKA inhibitor s1582 in primary hippocampal neuron. Decreased cell viability was significantly detectable in the group treated with 100μmol/L s1582 at 24h，(n=6). Data were expressed as mean ± SD, *P < 0.05 versus 50μM. Statistical details were provided in Supporting information 1.

**Supplementary Figure 9**

**
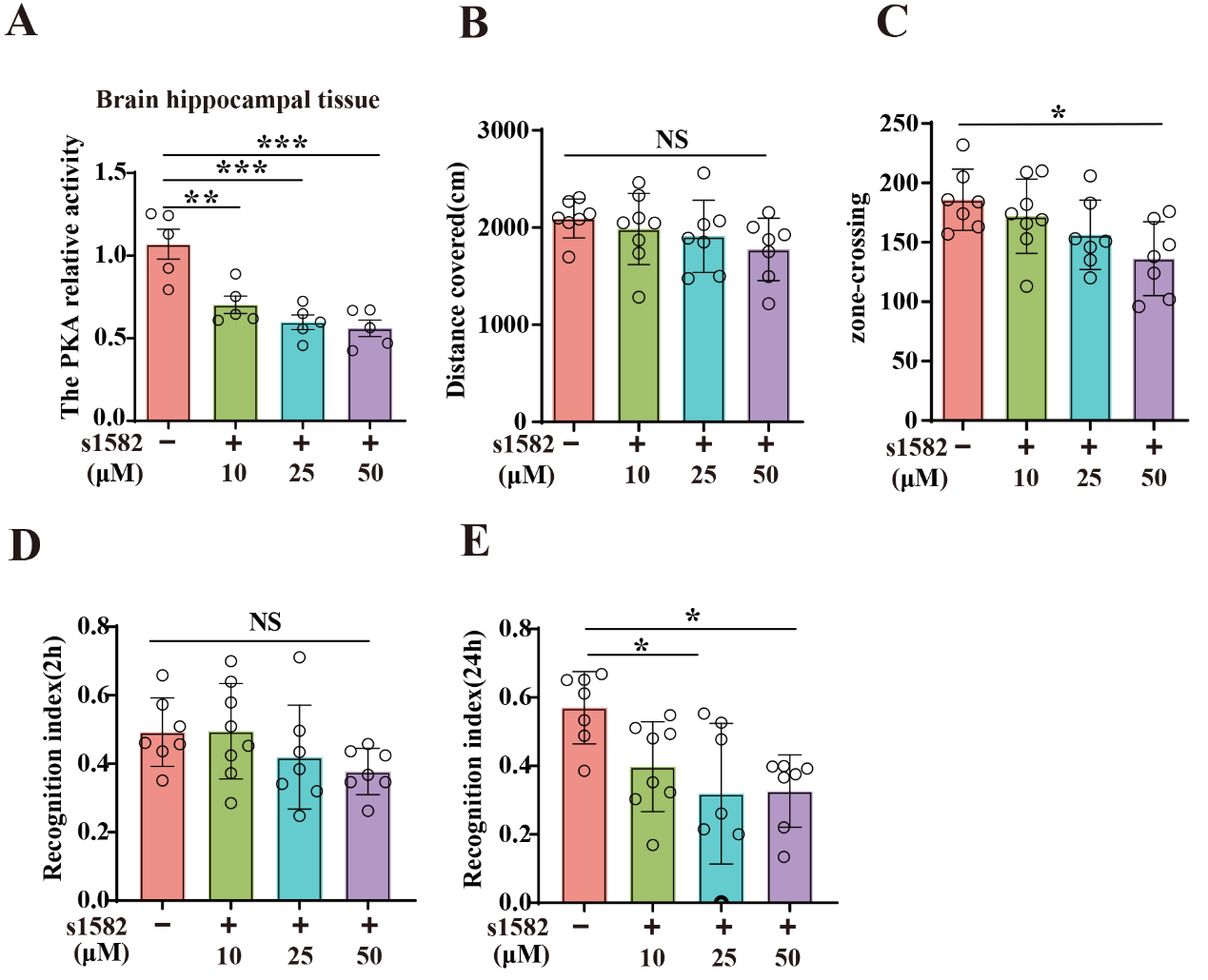
**

**sFigure 9 PKA inhibitor downregulates the CREB/SHANK1 pathway.**

1. By injection s1582 into hippocampus CA1, the PKA kinase activity was detected by Elisa Kit (n=5). (B, C) The open field test measured the total distance covered (B) and zone crossing (C) in the two groups, (n=7-8). The novel object recognition test showed the recognition index at 2hours (D) and at 24hours (E), (n =7-8). Data were expressed as mean ± SD, *P < 0.05, **P < 0.01, ***P < 0.001vs Control. Statistical details were provided in Supporting information 1.

**Supplementary Figure 10**

**
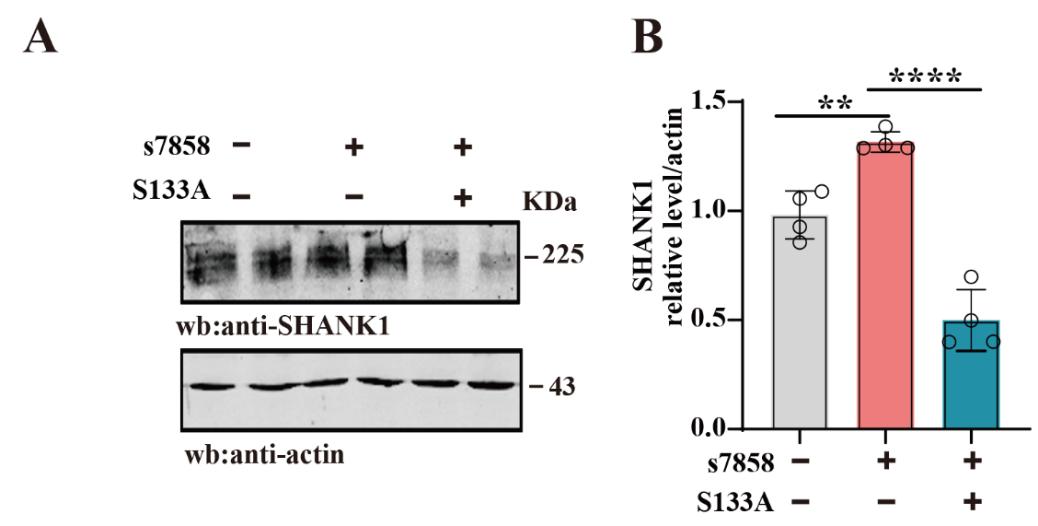
**

**sFigure 10 CREB is essential for PKA regulation of SHANK1 expression.**

(A) Western blotting was employed to detect the SHANK1 protein level, actin was used as a loading control. Quantitative analysis of the SHANK1, (n=4), (B) Data were expressed as mean ± SD, **P < 0.01, ****P < 0.0001vs s7858. Statistical details were provided in Supporting information 1.

**Supplementary Figure 11**

**
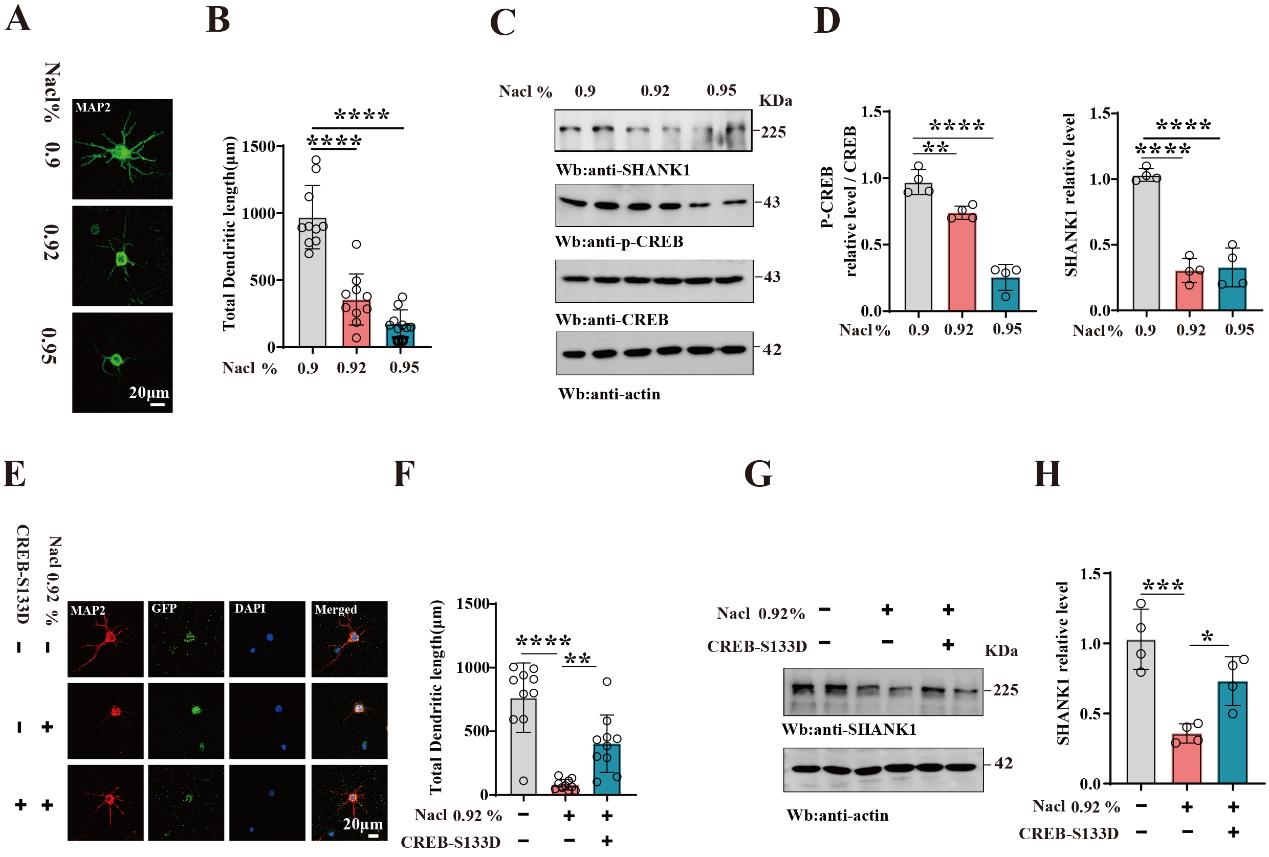
**

**sFigure 1****1 CREB-S133D could rescue the HS-induced SHANK1 deficiency and neuronal damage**

Rats’ primary hippocampal neuron were exposed to various concentrations (0.9%, 0.92%,0.95%) of NaCl for 0.5 h at 37°C to simulate a high-salt environment in vitro, representative images after treatment (A), (Scale bar = 20 μm), quantitative analyses of dendritic length (B), (n = 10 hippocampal neurons). (C) Western blotting was employed to detect the SHANK1, ps133-CREB/CREB protein levels, actin was used as a loading control. Quantitative analysis of the SHANK1, ps133-CREB/CREB (n=4), (D). Transfection of the mutant virus CREB-S133D under 0.92% NaCl conditions, representative images after treatment (E), (Scale bar = 20 μm), quantitative analyses of dendritic length (F), (n=10 hippocampal neurons). (G) Western blotting was employed to detect the SHANK1, actin was used as a loading control. Quantitative analysis of the SHANK1, (n=4), (H). Data were expressed as mean ± SD, *P < 0.5, **P < 0.01, ***P < 0.001, ****P < 0.0001vs Control or 0.92%. Statistical details were provided in Supporting information 1.
