# Supplementary material for: Long Term High‐Salt Diet Induces Cognitive Impairments via Down‐Regulating SHANK1
Source: Adv Sci (Weinh). 2025 Jun 26;12(36):e02099. doi: 10.1002/advs.202502099 (PMC12463032; doi:10.1002/advs.202502099)
Supplement: Supplementary file 3 — Supporting Information [file ADVS-12-e02099-s003.zip › SupplementaryMaterialS1.docx]

**Antibodies used in this study**

| Antibodies | | SOURCE | | IDENTIFIER | |
| --- | --- | --- | --- | --- | --- |
| Beta Actin | 1:2000 for WB | | Proteintech | | 66009-1-Ig |
| SHANK1 | 1:1000 for WB  1:200 for IF | | Abcam | | ab94576 |
| Phospho-CREB (Ser133) (87G3) | 1:1000 for WB, | | Cell signaling | | #9198 |
| CREB (48H2) | 1:1000 for WB | | Cell signaling | | #9197 |
| MAP2 | 1:300 for IF | | millipore | | AB5622 |
| CoraLite594 – conjugated Goat Anti-Mouse | 1:200 for IF | | Proteintech | | SA00013-3 |
| CoraLite488-conjugated Goat Anti-Rabbit IgG(H+L) | 1:200 for IF | | Proteintech | | SA00013-2 |
| CoraLite647-conjugated AffiniPure F(ab')2 Fragment Goat Anti-Rabbit IgG (H+L) | 1:200 for IF | | Proteintech | | SA00014-9 |
| HRP-conjugated Goat anti-Mouse IgG (H+L) | 1:10,000 for WB | | Abclonal | | AS003 |
| HRP-conjugated Goat anti-Rabbit IgG (H+L) | 1:10,000 for WB | | Abclonal | | AS014 |
| IRDye 800CW Goat anti-Mouse IgG | 1:10,000 for WB | | LI-COR Biosciences | | AB_621842 |
| IRDye 800CW Goat anti-Rabbit IgG | 1:10,000 for WB | | LI-COR Biosciences | | AB_621843 |

WB: Western blotting; IF: Immunofluorescence

**Chemicals used in this study**

| **REAGENT** | | **SOURCE** | | | **IDENTIFIER** |
| --- | --- | --- | --- | --- | --- |
| Cycloheximide | MCE | |  | HY-12320 | |
| DMEM/F12 | Gibco | |  | 11320082 | |
| Neurobasal | Gibco | |  | 21103049 | |
| B-27 supplement | Gibco | |  | 17504044 | |
| GlutaMAX Supplement | Gibco | |  | 35050061 | |
| 0.25% Trypsin (1X) | GIBCO | |  | 15050-065 | |
| Dual-Luciferase Reporter Assay System | Promega | |  | E1910 | |
| BCA Protein Assay Kit | Thermo | |  | 23227 | |
| Protease and phosphatase inhibitors | Thermo | |  | 78443 | |
| β-mercaptoethanol | Sigma-Aldrich | |  | 63689 | |
| Sodium dodecyl sulfate | Biosharp | |  | BS088 | |
| Bovine serum albumin | Solarbio | |  | A8020 | |
| Triton-X100 | Sigma-Aldrich | |  | 93443 | |
| Total RNA extraction kit | Solarbio | |  | R1200 | |
| 4×SDS-PAGE Concentrated Adhesive Buffer (pH6.8) | PUMOKE | |  | PMK0204 | |
| 4×SDS-PAGE Separation Glue Buffer (pH8.8) | PUMOKE | |  | PMK0205 | |
| Ammonium persulfate | PUMOKE | |  | PMK0562 | |
| 2×Universal SYBR Green Fast qPCR Mix | ABclonal | |  | RK21203 | |
| Penicillin/streptomycin | Gibco | |  | 15070063 | |
| Contains DAPI sealer | Beyotime | |  | P0131 | |
| CCK8 kit | Beyotime | |  | C0037 | |
| Simple ChIP® Plus Sonication Chromatin IP Kit | CST | |  | #56383 | |
| cAMP Assay test | Wuhan Baiyixin | |  | BK101 | |
| PKA Kinase Activity Kit | abcam | |  | ab139435 | |
| PKA inhibitor | Selleck | |  | s1582 | |
| PKA agonist | Selleck | |  | s7858 | |
