# Supplementary material for: Long Term High‐Salt Diet Induces Cognitive Impairments via Down‐Regulating SHANK1
Source: Adv Sci (Weinh). 2025 Jun 26;12(36):e02099. doi: 10.1002/advs.202502099 (PMC12463032; doi:10.1002/advs.202502099)
Supplement: Supplementary file 3 — Supporting Information [file ADVS-12-e02099-s003.zip › SupplementaryMaterialS2.docx]

The primers for the target genes SHANK1 for ChIP-qPCR were designed as follows.

| 1 | 1F CACCCTGGTCTCCAACCACTC  1R AAAGAATAGCCCTGGGTCGG | 209bp |
| --- | --- | --- |
| 2 | 2F CTCTCCCGACCCAGGGCTAT  2R GCTAAGAATAGCTGGCACGCA | 214bp |
| 3 | 3F CCAGCTGCGTGCCAGCTAT  3R GGCTGACATCCCCTTTGCAG | 223bp |
| 4 | 4F GGGCTGCAAAGGGGATGTC  4R GAGCAGGCGTCTAACCACGG | 225bp |
| 5 | 5F GTTAGACGCCTGCTCGCCTA  5R CCCCTACTCCCCACTCTAGAACC | 226bp |
| 6 | 6F TCCGGTTCTAGAGTGGGGAGTA  6R CCACCGAGGAGAATTTGGATT | 189bp |
| 7 | 7F ACCCAATCCAAATTCTCCTCG  7R GGTTACTGGGGCGTGTGAAGA | 209bp |
| 8 | 8F CACCCACACATCCTCTTCACAC  8R AGCAGGAGGTGCCCGAGAG | 207bp |
| 9 | 9F ACACTTCAGTGAAGTCACCAGCTC  9R AAAATGAAGCTAAAATTAGCTAAGGAC | 195 bp |
| 10 | 10F CTGTCCTTAGCTAATTTTAGCTTCATT  10R TGCACAGACAAGTTTGCACCA | 202 bp |
| 11 | 11F GCAGAGGTGGTGCAAACTTGT  11R GACAGCAGCATCACAGGCAG | 155 bp |
