# Supplementary material for: Long Term High‐Salt Diet Induces Cognitive Impairments via Down‐Regulating SHANK1
Source: Adv Sci (Weinh). 2025 Jun 26;12(36):e02099. doi: 10.1002/advs.202502099 (PMC12463032; doi:10.1002/advs.202502099)
Supplement: Supplementary file 3 — Supporting Information [file ADVS-12-e02099-s003.zip › SupplementaryMaterialS3.pptx]

## Slide 1
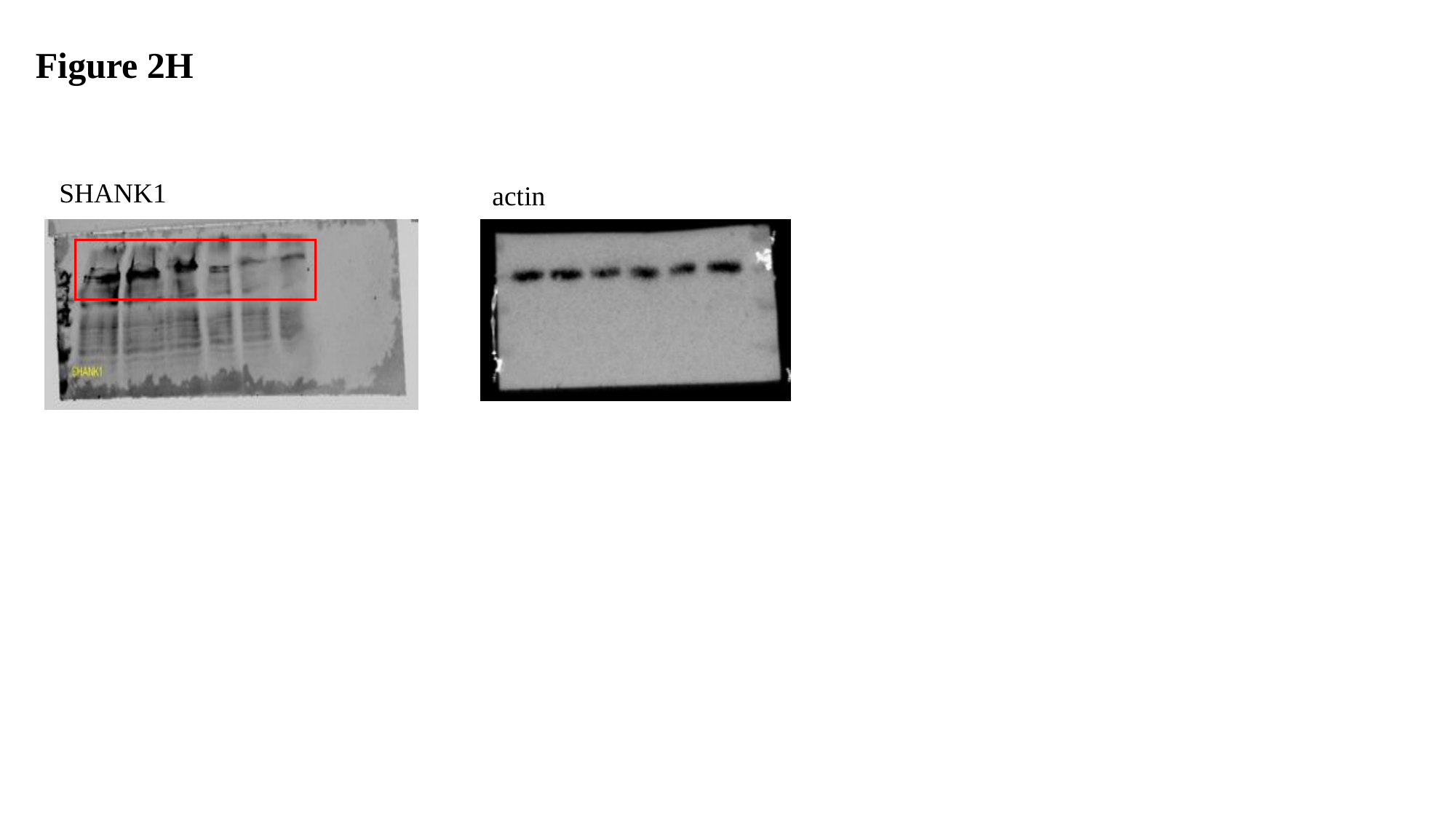

Figure 2H
SHANK1
actin

## Slide 2
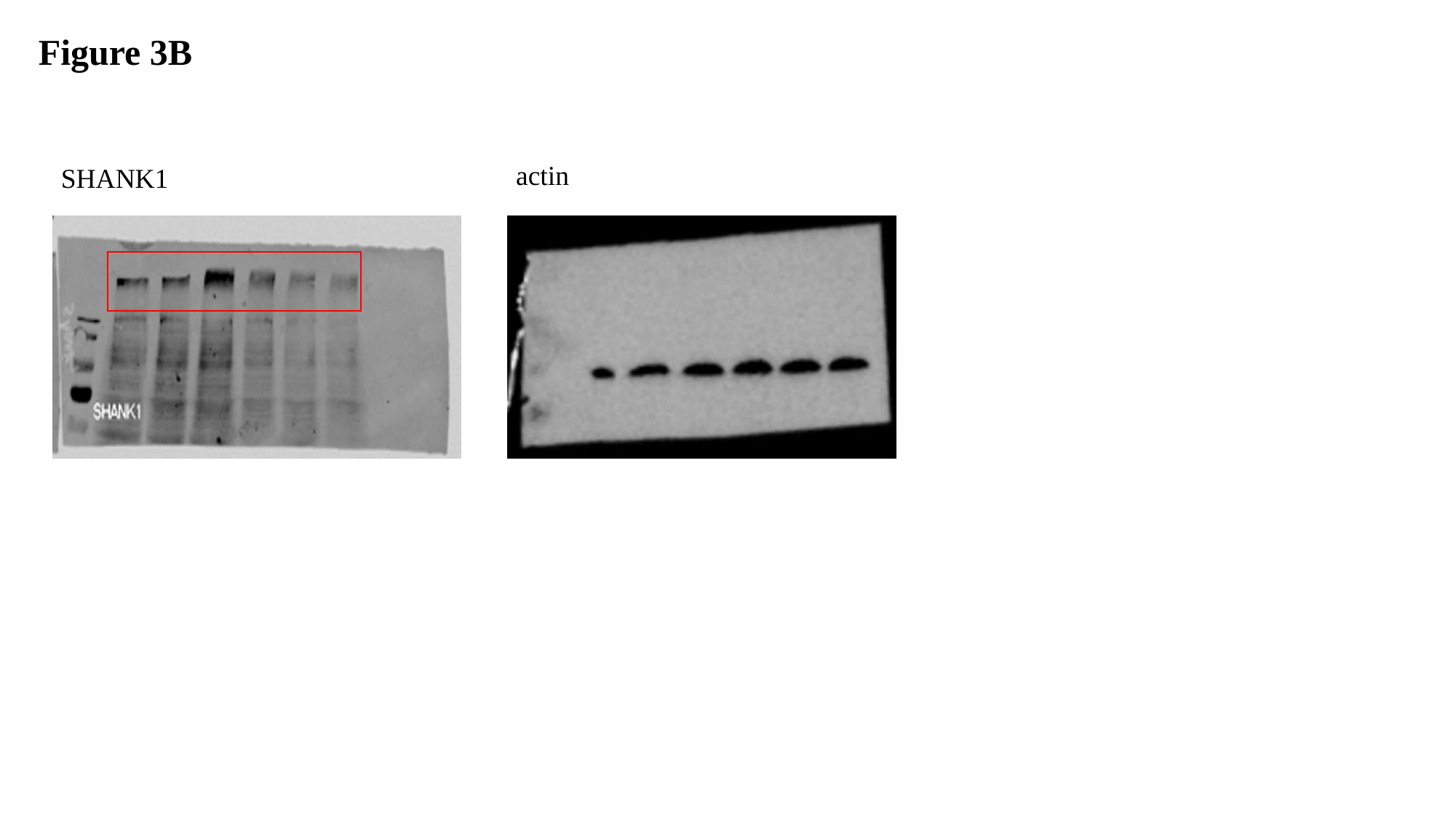

Figure 3B
actin
SHANK1

## Slide 3
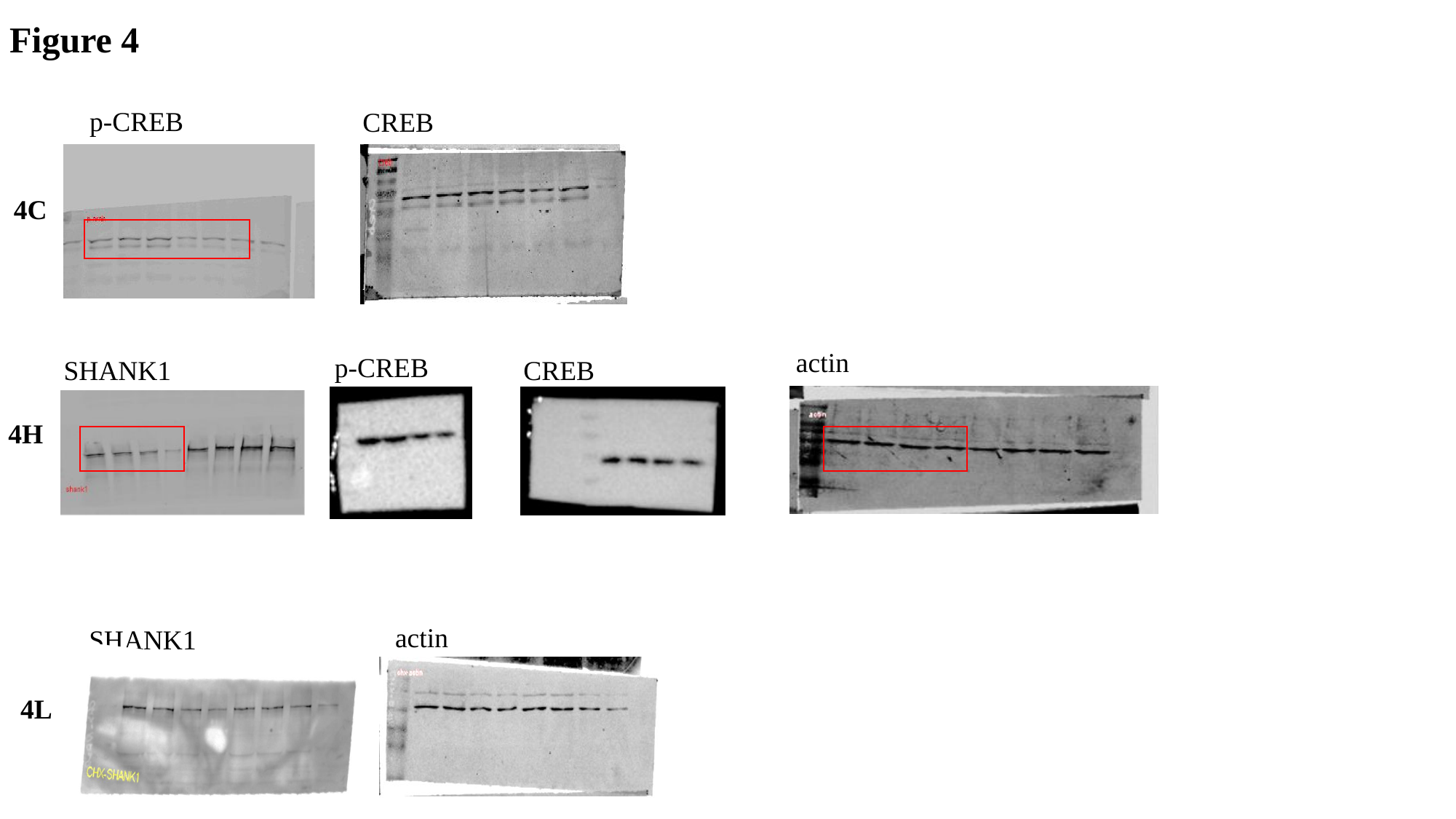

Figure 4
p-CREB
CREB
4C
actin
p-CREB
CREB
SHANK1
4H
actin
SHANK1
4L

## Slide 4
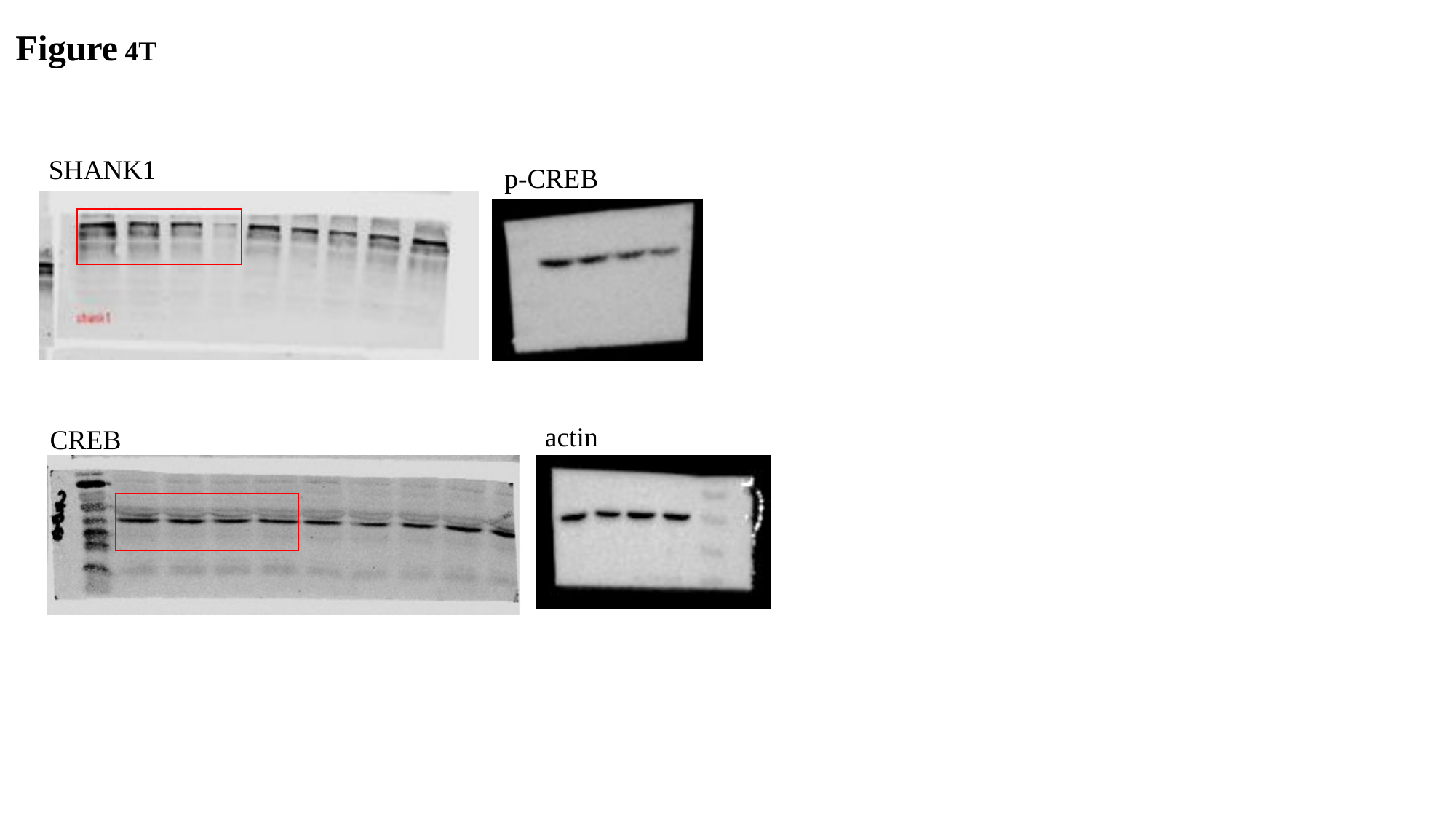

Figure 4T
SHANK1
p-CREB
actin
CREB

## Slide 5
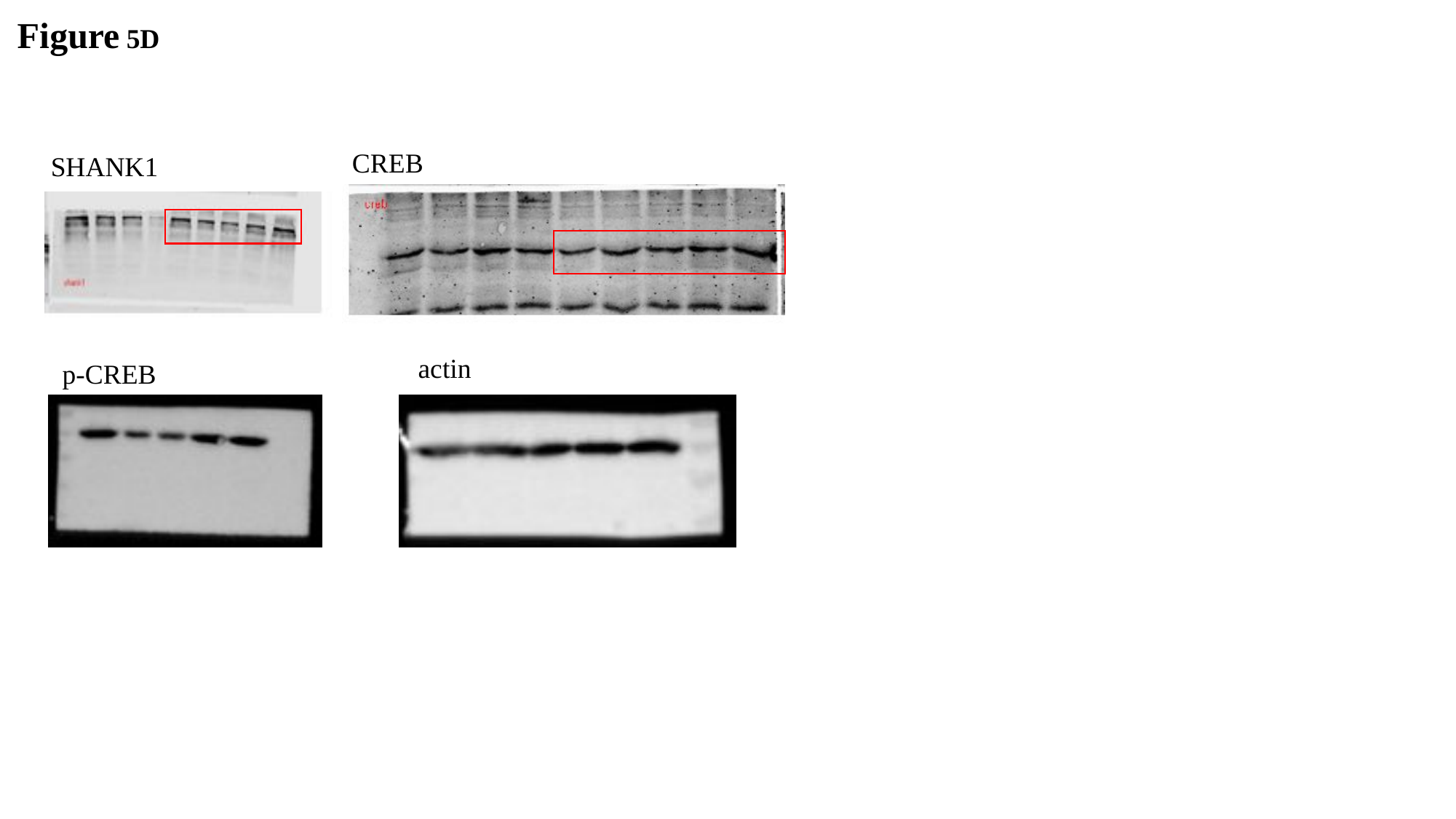

Figure 5D
CREB
SHANK1
actin
p-CREB

## Slide 6
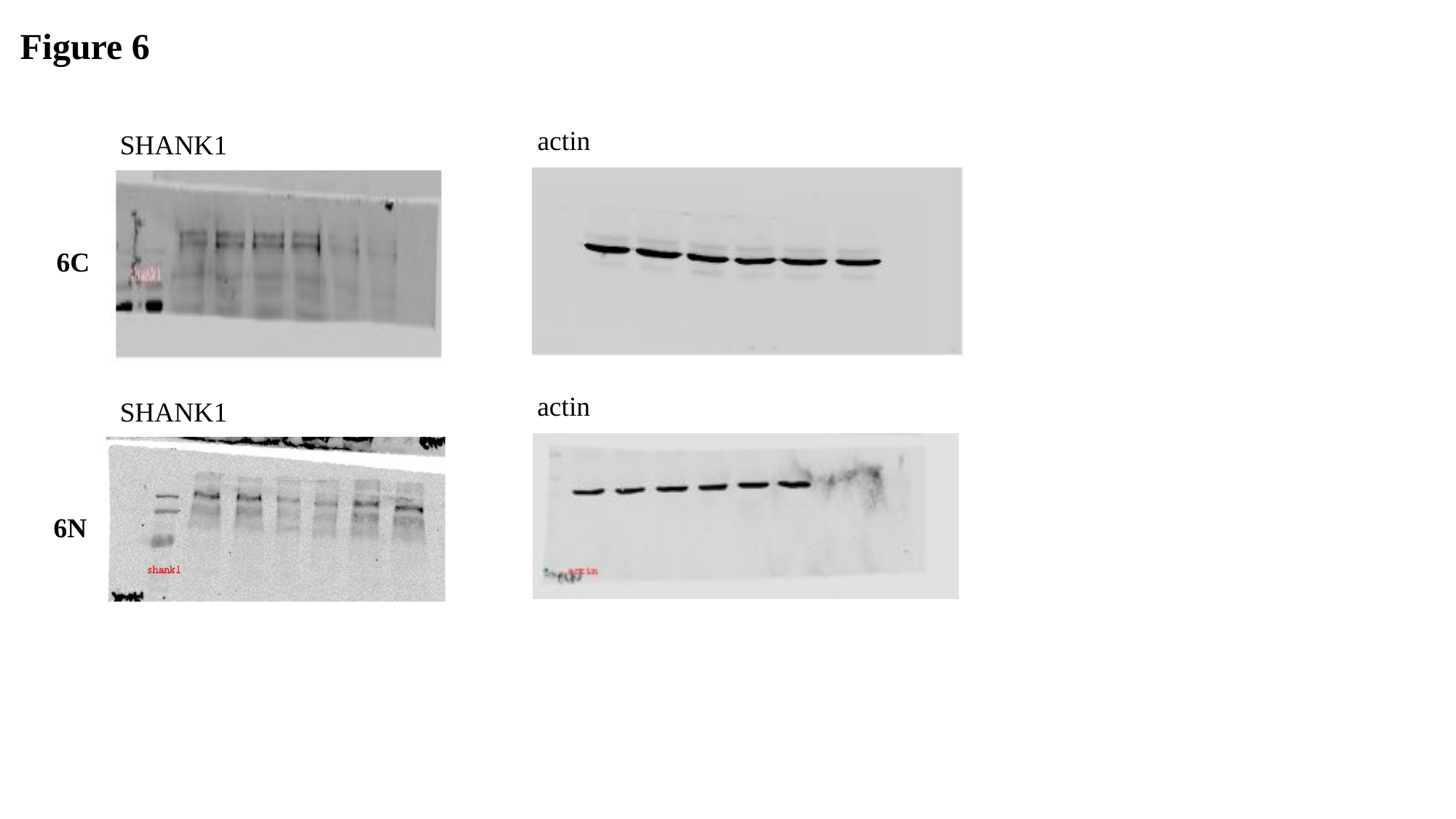

Figure 6
actin
SHANK1
6C
actin
SHANK1
6N

## Slide 7
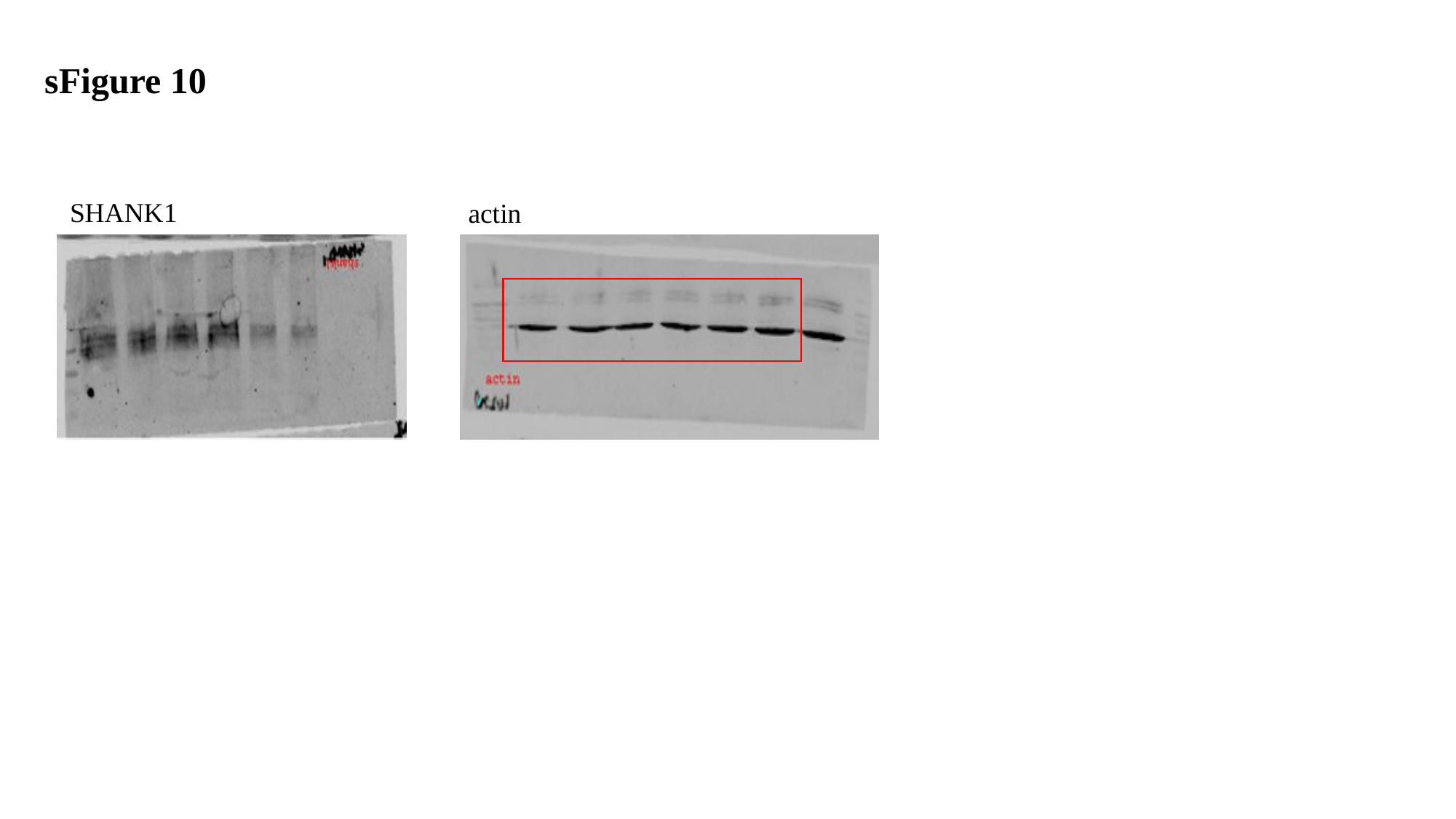

sFigure 10
SHANK1
actin

## Slide 8
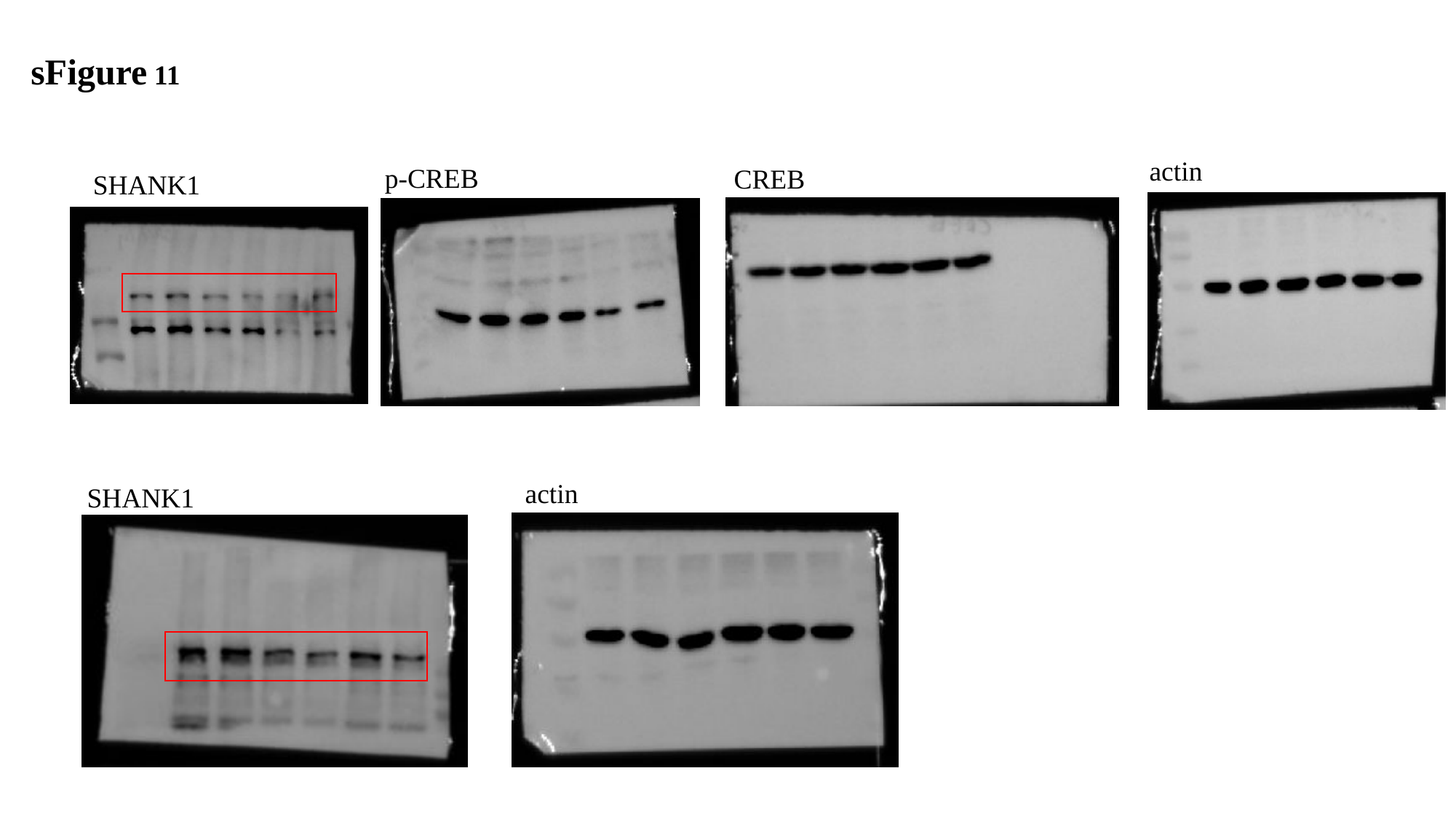

sFigure 11
actin
p-CREB
CREB
SHANK1
actin
SHANK1
